# Metastatic disease and major adverse cardiovascular events preceding diagnosis are the main determinants of disease-specific survival of pheochromocytoma/paraganglioma: long-term follow-up of 303 patients

**DOI:** 10.3389/fendo.2024.1419028

**Published:** 2024-08-21

**Authors:** Wolfgang Raber, Raphael Schendl, Melisa Arikan, Andreas Scheuba, Peter Mazal, Valerie Stadlmann, Reinhard Lehner, Petra Zeitlhofer, Sabina Baumgartner-Parzer, Cornelia Gabler, Harald Esterbauer

**Affiliations:** ^1^ Department of Medicine III, Clinical Division of Endocrinology and Metabolism, Medical University of Vienna, Vienna, Austria; ^2^ Department of Surgery, Medical University of Vienna, Vienna, Austria; ^3^ Department of Clinical Pathology, Medical University of Vienna, Vienna, Austria; ^4^ Department of Medical Genetics, Medical University of Vienna, Vienna, Austria; ^5^ Labdia Labordiagnostik, and St. Anna Children’s Cancer Research Institute (CCRI), Vienna, Austria; ^6^ Department of IT Systems and Communications, Medical University of Vienna, Vienna, Austria; ^7^ Department of Laboratory Medicine, Medical University of Vienna, Vienna, Austria

**Keywords:** pheochromocytoma, paraganglioma, recurrence, survival, genetics, natural history, long-term follow-up

## Abstract

**Purpose:**

The natural history in unselected cohorts of patients with pheochromocytoma/ paraganglioma (PPGL) followed for a period >10 years remains limited. We aimed to describe baseline characteristics and outcome of a large cohort and to identify predictors of shorter survival.

**Methods:**

This retrospective single-center study included 303 patients with newly diagnosed PPGL from 1968 to December 31, 2023, in 199 prospectively supplemented since July 2020. Mean follow-up was 11.4 (range 0.3-50) years, germline genetic analyses were available in 92.1%. The main outcome measures were overall (OAS), disease-specific (DSS), recurrence-free (RFS) survival and predictors of shorter survival evaluated in patients with metastases at first diagnosis (n=12), metastatic (n=24) and nonmetastatic (n=33) recurrences and without evidence of PPGL after first surgery (n=234).

**Results:**

Age at study begin was 49.4 ± 16.3 years. There were 72 (23.8%) deaths, 15 (5.0%), 29 (9.6%) and 28 (9.2%) due to PPGL, cardiovascular disease (CVD) and malignant or other diseases, respectively. Median OAS, DSS1 (tumor-related) and DSS2 (DSS1 and death caused by CVD) were 4.8, 5.9 and 5.2 years (patients with metastases at first diagnosis), 21.2, 21.2 and 19.9 years, and 38.0, undefined and 38.0 years (patients with metastatic and with nonmetastatic recurrences, respectively). Major adverse cardiovascular events (MACE) preceded the first diagnosis in 15% (n=44). Shorter DSS2 correlated with older age (P ≤ 0.001), male sex (P ≤ 0.02), MACE (P ≤ 0.01) and primary metastases (P<0.0001, also for DSS1).

**Conclusion:**

The clinical course of unselected patients with PPGL is rather benign. Survival rates remain high for decades, unless there are MACE before diagnosis or metastatic disease.

## 1 Introduction

Pheochromocytoma (PCC) and paraganglioma (PGL), together PPGL, are rare neuro-endocrine tumors occurring with an annual incidence of 0.04-0.66 per 100.000 individuals (1, 2). These tumors have the highest degree of heritability of all human neoplasias. Approximately 40% of patients harbor a pathogenic germline mutation in one of the about 20 driver genes discovered so far (3). Discovery may be either incidental, due to screening procedures or because of adrenergic symptoms. Many patients with PPGL may in fact have symptoms suggestive of catecholamine excess, yet these may remain unrecognized by patients and/or physicians even for years (4, 5). Clinical presentation of PPGL with life-threatening major cardiovascular events (MACE) are dramatic challenges to patients and physicians (6–10). The prognostic impact to long-term prognosis of different modes of clinical presentation is not known, however.

Knowledge of the natural history of PPGL in unselected cohorts followed for a period >10 years remains limited. Most studies report overall survival (OAS). The primary measure of interest, the disease-specific survival (11), has, however, rarely been assessed and if so, cohorts were either highly selected or included patients from multiple centers of many countries and definitions of ´disease-specific´ were not uniform (12–14). Reported independent prognostic factors of survival differ greatly, and no study has included the mode of presentation, especially the occurrence of MACE prior to first diagnosis, in the survival analyses (12–19).

We report a cohort of 303 patients with newly diagnosed PPGL followed for up to 50 years and aimed to assess detailed baseline characteristics, OAS, disease-specific survival (DSS) and recurrence-free (RFS) survival, in addition to predictors of shorter survival in patients with primary metastases (n=12), metastatic (n=24) and nonmetastatic (n=33) recurrences, as well as without evidence of PPGL after first surgery (n=234). At least one molecular genetic analysis for germline variants associated with hereditary disease was available in 279 patients (92.1%) - in 153 (50.5% of the total cohort) by state-of-the-art next generation sequencing (NGS). In July 2020, the study was approved by the Ethics Board of the Medical University of Vienna (Nr. 1022/2020).

## 2 Materials and methods

### 2.1 Patients

A thorough search of the electronic patient management system of the Vienna General Hospital for the years 1992 to 2023 and of the electronic coding system of the Clinical Division of Endocrinology & Metabolism, Department of Medicine III of the Vienna General Hospital for the years 1968 to 2017, were performed. The former database covers all patients admitted to the Vienna General Hospital, the latter all visits to the out-patient unit of the Division of Endocrinology. The database query was for Codes of the International Classification of Disease Version 10 and 9 (ICD-10 and ICD-9) with known association to PPGL that had been entered into PDF documents of the discharge and surgical reports (Details in the **Supplementary Material**). Inclusion criteria were either, histopathological proof of PPGL after surgery (n=299) or the diagnosis of PPGL by (18) Fluoro-dihydrophenylalanine (F-DOPA) positron-emission tomography (PET)-CT (n=3) or biopsy (n=1) in four multimorbid patients refusing surgery. Patients treated for a malignant comorbidity at the time of first diagnosis of PPGL were excluded.

**Figure 1:**
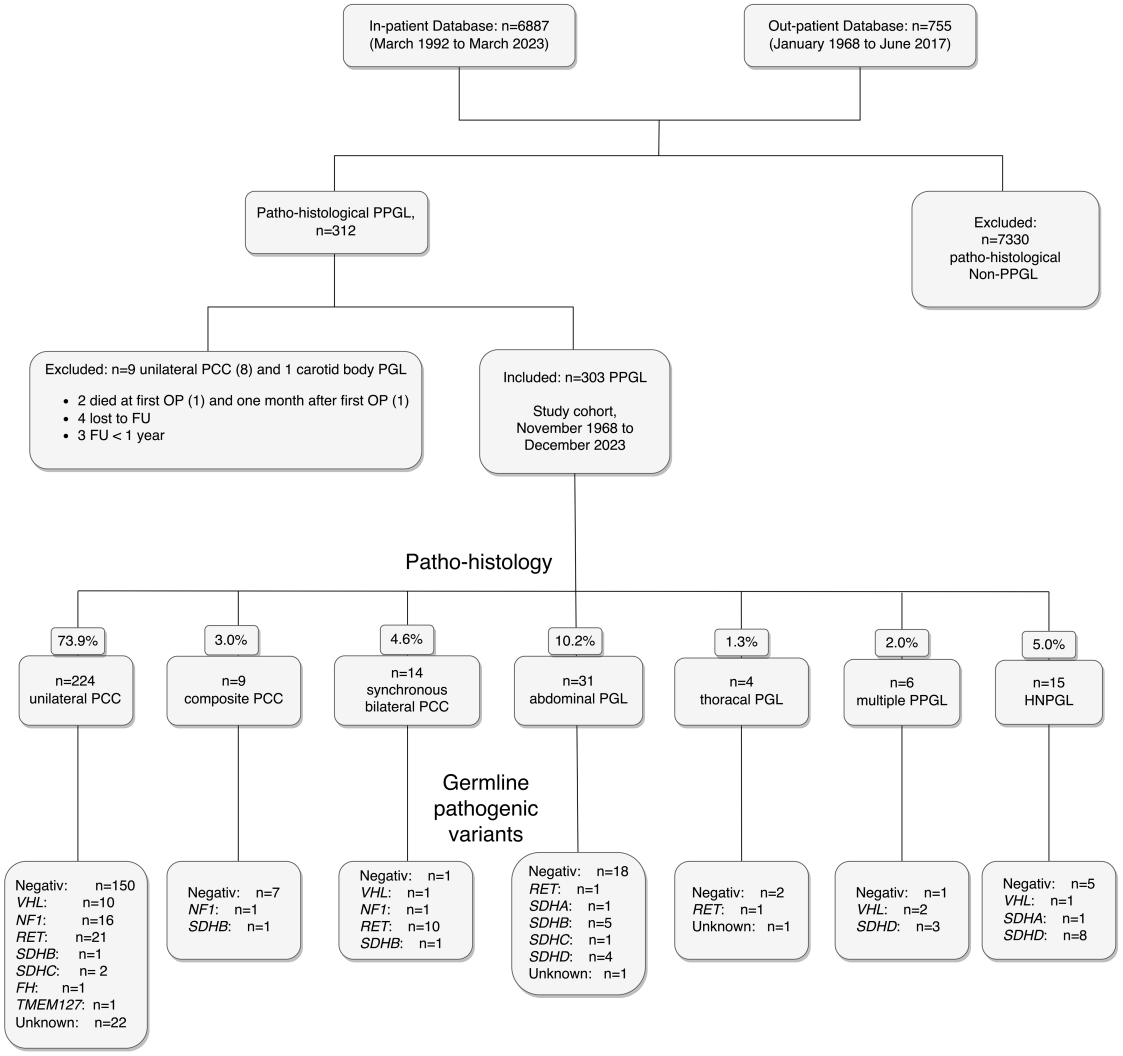
Flowchart of the study cohort of 303 patients with PPGL, including histopathological diagnoses and pathogenic germline genetic findings. Abbreviations are those used in the text.

The first systematic search of the Austrian Death Registry in July 2020 identified 58 (19.1%) deceased patients (data current until December 31, 2019). According to Austrian law, health data after death may be used for scientific purpose without informed consent. Thus, the data of these 58 deceased patients were included in the analyses. All survivors by January 1, 2020 were then invited for further prospective evaluation, which 199 patients (81.2%) accepted. All 199 have since been seen on a regular out-patient basis by one of the authors (W.R.) until today. The second consultation of the Austrian Death Registry in October 2023 (data current through December 31, 2022) resulted in additional ten nonsurvivors. The remaining 32 patients were contacted by telephone and four additional deaths were identified. The other 28 of these 32 patients refused to present for FU examination, but missing data of their medical history could be collected. By the end of the study, there were 72 nonsurvivors (23.8%) and 231 (76.2%) survivors. One hundred fifty-nine (68.8%) survivors were last seen in 2023, an additional 40 (17.3%) patients in 2022. A flowchart of the study cohort is shown in **Figure 1**.

### 2.2 Methods

#### 2.2.1 Clinical presentation and comorbidities

Nine clinical scenarios leading to the diagnosis of PPGL were identified in 291 (96.0%) patients: (a) incidental diagnosis during imaging or laboratory procedures performed for reasons unrelated to PPGL, (b) screening in the context of familial syndromes, (c) adrenergic symptoms (at least two of the typical triad of paroxysmal headache, palpitations and diaphoresis), diagnostic work-up for (d) uncontrolled or (e) suspected secondary hypertension, (f) signs and symptoms (growing lump in the neck, hearing problems) suggestive of head-and-neck PGL (HNPGL), (g) incidental despite MACE within five years preceding the first diagnosis of PPGL, (h) uncontrolled hypertension despite MACE within five years preceding the first diagnosis and (i) MACE leading to the diagnosis (**Table 1**). For the purposes of this study, MACE were defined as a life threatening event of the cerebrovascular, cardiovascular or peripheral arterial system including transient ischemic attacks (TIA), prolonged reversible ischemic deficits (PRIND), ischemic or hemorrhagic stroke, acute coronary syndrome (ACS), non ST-elevation myocardial infarction (NSTEMI), ST-elevation myocardial infarction (STEMI), non-specified acute MI (AMI), dissecting aortic aneurysm or critical peripheral artery ischemia.

**Table 1:** Characteristics of the study cohort (n=303 PPGL) followed for up to 50 years according to survivors (n=231) and nonsurvivors (n=72).

	All (n=303)	Survivors (n=231)	Non-survivors (n=72)	p-value
Age at 1st surgery in years, mean ± SD	49.4 ± 16.3	46.7 ± 15.3	58.0 ± 16.6	<0.0001
>49a, n (%)	156 (51.5)	105 (45.5)	51 (70.8)	0.0002
Female sex, n (%)	167 (55.1)	135 (58.4)	32 (44.4)	0.04
Histopathological diagnosis				0.26
PCC group, all, n (%)	247 (81.5)	190 (82.3)	57 (79.2)	
uPCC unilateral, n (%)	224 (73.9)	173 (74.9)	51 (70.8)	
cPCC unilateral, n (%)	9 (3.0)	6 (7.5)	3 (4.2)	
bPCC synchronous, n (%)	14 (4.6)	11 (4.8)	3 (4.2)	
PGL group, all, n (%)	41 (13.5)	28 (12.1)	13 (18.1)	
aPGL, n (% PGL not HNPGL)	31 (88.6)	21 (95.5)	10 (76.9)	
tPGL, n (% PGL not HNNPGL)	4 (11.4)	1 (4.5)	3 (23.1)	
mPPGL synchronous	6 (2.0)	6 (2.6)	0	
HNPGL	15 (5.0)	13 (5.6)	2 (2.8)	
Clinical scenarios at presentation				0.0004
Oligosymptomatic group, n (%)	103 (34.0)	84 (36.4)	19 (26.4)	
a) Incidental, n (%)	89 (29.4)	71 (30.7)	18 (25.0)	0.65 (incidental vs. all other)
≥2 classical symptoms, n (% incidental)	13 (14.6)	11	2	
Weight loss, n	5	4	1	
Abdominal or back pain, n	5	2	3	
Hypertension, n	21	17	4	
Really no symptoms, n	45	37	8	
b) Screening, n (%)	14 (4.6)	13 (5.6)	1 (1.4)	0.21 (all screening vs. all other presentations)
≥2 classical symptoms, n	1	1	0	
Weight loss, n	1	1	0	
Hearing problems, n	1	1	0	
Really no symptoms, n	12	11	1	
Non-MACE symptomatic group, n (%)	144 (47.5)	119 (51.5)	25 (34.7)	0.004 (adrenergic sympt vs. all other pres)
c) Adrenergic symptoms, n (%)	96 (31.7)	84 (36.4)	12 (16.7)	0.59 (uncontr hypertens vs. all other pres)
d) Uncontrolled hypertension, n (%)	22 (7.3)	16 (6.9)	6 (8.3)	0.34 (susp of 2nd hypertens vs. all other pres)
e) Suspicion of secondary hypertension, n (%)	15 (5.0)	10 (32.2)	5 (6.9)	0.0003 (MACE leading to dx vs. all other pres)
f) Lump in the neck/hearing problems, n (%)	11 (3.6)	9 (3.9)	2 (2.8)	
MACE group, all, n (%)	44 (14.5)	23 (10.0)	21 (29.2)	0.19 (MACE not leading to dx vs. all other pres)
g) Incidental despite prior MACE, n (%)	22 (7.3)	14 (6.1)	8 (11.1)	<0.0001 (all MACE vs. all other presentations)
h) Uncontr hypertension desp prior MACE, n (%)	4 (1.3)	2 (0.8)	2 (2.8)	0.0005 (MACE vs. asympt)
k) MACE, n (%)	18 (5.9)	7 (3.0)	11 (15.3)	0.0001 (MACE vs. non-MACE sympt)
Unknown, n (%)	12 (4.0)	5 (2.2)	7 (9.7)	0.87 (Asympt vs. non-MACE sympt)
Comorbidities				<0.0001
No comorbid disease group, n (%)	151 (49.8)	135 (58.4)	16 (22.2)	
No comorbid disease, n (%)	44 (14.5)	37 (16.0)	7 (9.7)	<0.0001 (No comorb. vs all comorb comb)
≥ 2CV risk factors (CV RF), n (%)	107 (35.3)	98 (42.4)	9 (12.5)	<0.0001 (≥ 2CV RF vs. all comorb comb)
CV disease (CVD), n (%)	81 (26.7)	50 (21.6)	31 (43.1)	0.86 (CVD vs. other comorbidities)
Malignancy dominated group, n (%)	52 (17.2)	38 (16.5)	14 (19.4)	0.02 (CVD vs. all other comb)
Malignant disease without CV RF, n (%)	22 (7.3)	15 (6.5)	7 (9.7)	0.02 (Mal+CVD vs. Mal dom)
Malignant disease with CV RF, n (%)	19 (6.3)	14 (6.1)	5 (6.9)	
Other*, n (%)	11 (3.6)	9 (3.9)	2 (2.8)	0.13 (CVD vs. Mal+CVD)
Malignant disease +CVD, n (%)	19 (6.3)	8 (3.5)	11 (15.3)	0.19 (CVD vs. Mal dom)
Genetic results				<0.0001
Positive, n (%)	95 (31.4)	76 (32.9)	19 (26.4)	<0.0001 (pos vs. unknown)
Cluster 1A, n (% of positive)	29 (30.2)	25 (33.3)	4 (21.1)	<0.0001 (neg vs. unknown)
Cluster 1B, n (% of positive)	14 (14.6)	14 (18.7)	0	0.88 (pos vs. neg)
Cluster 2, n (% of positive)	52 (55.2)	37 (48.7)	15 (78.9)	
Negative, n (%)	184 (60.7)	148 (64.1)	36 (50.0)	0.02 (Cluster 2 vs. Cluster 1)
Unknown, n (%)	24 (7.9)	7 (3.0)	17 (23.6)	
Death in the 1990s, 2000s, 2010s, 2020s, n			6, 7, 2, 2	
Tumor size in cm, mean ± SD	5.3 ± 2.8	4.8 ± 2.3	6.9 ± 3.5	<0.0001
<6cm, n (% of data available)	186 (65.3)	157 (71.4)	29 (44.6)	0.0003
≥6cm, n (% of data available)	99 (34.7)	63 (28.6)	36 (55.4)	
Primary metastatic, n (%)	12 (4.0)	1 (0.4)	11 (15.3)	<0.0001
of which local Lnn., n (%)	6 (50.0)	1 (100.0)	5 (45.5)	
of which distant sites, n (%)	6 (50.0)	0	6 (54.5)	

Cluster 1A included pathogenic germline variants of succinate dehydrogenase subunits A-D (SDHA-D) and fumarate hydratase (FH), cluster 1B of von Hippel-Lindau (VHL) tumor suppressor gene and cluster 2 of rearranged-during-transfection (RET) proto-oncogene, neurofibromin 1 (NF1) tumor suppressor gene and transmembrane protein 127 (TMEM127). More detailed characteristics of patients are given in **Supplementary Table 4**. *Details of “other” comorbidities are given in **Supplementary Table 3**.

To allow sufficient power for the Cox regression analyses, the nine parameters were combined to establish three groups: all MACE together (the MACE group), patients with adrenergic symptoms, uncontrolled hypertension or the suspicion of secondary hypertension (the group of non-MACE symptoms) and patients diagnosed incidentally or due to screening procedures (the oligosymptomatic group). The MACE group included both MACE before first diagnosis and during FU, provided plasma metanephrines (P-MNs) or 24-hour unrinary metanephrines (U-MNs) were diagnostic of recurrence of PPGL (20) around the time of MACE. Of note, incidental diagnosis did not mean, that these patients were asymptomatic. The number of incidentally diagnosed patients suffering from various symptoms that had gone unnoticed to patients and/or physicians or those with MACE were significant and are detailed in **Supplementary Tables 1** and **2**, respectively.

The following comorbidities were assessed for all 303 patients: no symptoms; at least three of the following typical cardiovascular (CV) risk factors (RF): type-2 diabetes, hypertension, hypercholesterolemia, smoking or obesity; patients with established CVD or pulmonary disease, including atrial fibrillation or chronic obstructive pulmonary disease (COPD); patients with malignant disease; with malignant disease and CV RF; with malignant disease and concurrent CVD together; and patients with other illnesses (**Table 1**). Stage I tumors according to the TNM classification (21) were not considered malignant comorbidities. The detailed comorbidities of patients are given in **Supplementary Table 3**.

For the Cox regression, the comorbidity parameters were grouped as follows: no comorbidities and patients with CV RFs together (the non-disease group), patients with CVD or COPD as outlined above (the CVD group), malignancies with or without CV RF including other illness (the malignancy dominated group) and patients with malignancies and concurrent CVD, suspected to represent patients with the most serious comorbidities. For the prognostic model of the 291 patients with nonmetastatic disease at the begin of the study, the first two (the non-disease and the CVD) groups of patients and the latter two (those with malignant comorbidities) were considered together, respectively.

#### 2.2.2 Biochemical evaluation and imaging

Preoperative biochemical test results were available for 259 patients (85.5%). U-MNs were determined in 159 (52.5%), P-MNs in 132 (43.6%) and simultaneously in 112 (37.0%). Current assays for the measurements of U-MNs and P-MNs in our hospital were described previously (22). Further details as to biochemical testing and imaging procedures are described in the **Supplementary Material** (**Supplementary Text** and **Supplementary Table 4**).

#### 2.2.3 Surgery, histopathological diagnosis, PASS- and GAPP scores

The surgical approach to the first operation was available for 299 (98.7%) patients. Four multimorbid patients diagnosed with PPGL by F-DOPA PET-CT (n=3) or biopsy (n=1) refused surgery but were included in the analyses.

For 285 patients (94.1% of the total cohort) with histopathological confirmation of PPGL, the surgical specimens were examined by pathologists of the Vienna General Hospital with extensive experience in the diagnosis of neuro-endocrine tumors, for 14 patients (4.6%) the examination was performed elsewhere. The histopathological diagnoses were unilateral PCC (uPCC), unilateral composite PCC (PCC with variable proportions of ganglioneuroma cells, cPCC), synchronous bilateral PCC (bPCC), abdominal and intrathoracic PGL (aPGL and tPGL), synchronous multiple PGL (mPPGL) with (n=3) or without (n=3) PCC and HNPGL (**Figure 1**). Bilateral PCC was defined by either, tumorous lesions in both adrenals (n=12) or a unilateral tumor with contralateral diffuse or nodular hyperplasia of the adrenal medulla (n=2) (23). Occurrence was defined as synchronous, when PPGL were diagnosed simultaneously or within three months of each other and as recurrent PPGL, when there were more than three months in between the diagnoses. Twelve patients (3.4% of the study cohort) had primary metastatic PPGL. The remaining 291 patients were disease free after the first operation, as assessed by postoperative biochemical results within the reference range (n=245), by normal postoperative imaging (n=25) or both (n=31). For the Cox regression, patients with uPCC, cPCC and bPCC (the PCC group) and those with aPGL, tPGL and mPGL with or without PCC (the PGL group) were combined to create 3 variables (PCC, PGL and HNPGL) (**Table 1**). Pheochromocytoma of the Adrenal gland Scaled Score (PASS) was available for 214 (80.6%) patients, the Grading for Adrenal Pheochromocytoma and Paraganglioma (GAPP) score for 73 (24.1%). Patients were assessed for PASS score <4 and ≥4 points and for GAPP score <3, 3-6 and ≥7 points, equivalent to suggested low and high risk (PASS), and low, medium and high risk (GAPP) for metastatic recurrence, respectively (24, 25).

#### 2.2.4 Family history and germline genetic analysis (**Table 1**)

##### 2.2.4.1 Family history

Family history was available for 277 patients (91.4%). Great attention was paid to assess diseases related to multiple endocrine neoplasia type 2A (MEN-2A), von Hippel-Lindau syndrome (VHL), neurofibromatosis type 1 (NF1) and familial PGL syndromes. Family history was considered positive when patients reported either the established diagnosis of PPGL or an associated genetic syndrome in their family. Premature CVD or symptoms suggestive of PPGL in relatives were not considered positive family history.

##### 2.2.4.2 Germline genetic testing

Germline genetic analyses were performed in 279 patients (92.1%) of our study cohort. Four additional patients (1.3%) with NF-1 were diagnosed on clinical grounds (26). During the study period, there have been four different laboratories involved, two between the mid-1990s and mid-2010s responsible for the germline analyses of RET and of RET, SDHB, SDHC, SDHD, VHL, respectively, and two currently performing NGS and the neuroendocrine gene panel, respectively (**Supplementary Table 5**). More than 100 patients had NGS testing complementary to their sequencing of RET, SDHB, SDHC, SDHD and VHL genes in the past. Currently, NGS results are available for 153 patients (50.5%) of the cohort (in 126 simultaneously with neuroendocrine gene panel analyses), with the trend increasing. The close cooperation and regular boards between the endocrinologist (W.R.) and the genetic specialists (H.E., V.St. and R.L.) ensured best possible clinical interpretation of identified variants, leading to additional in-depth analyses upon availability of new clinical information. For the Cox regression, pathogenic genetic variants of individual genes are considered within distinct clusters (cluster 1A, 1B and 2) according to current definitions (27). The genetic methods used are described in detail in the **Supplementary Text**.

### 2.3 Statistics

Categorical variables are presented using number (%) of subjects, continuous data as mean ± SD or as median (range) depending on data type and distribution. Whenever possible, comparisons were made with the Chi Square test, the Fishers´ exact test, the one-sided t-test, the Mann-Whitney U test, one-way analysis of variance (ANOVA) followed by Tukey´s multiple comparisons test or the Kruskal-Wallis test followed by Dunn´s multiple comparisons test, as appropriate.

The length of FU for the OAS was defined as the number of years from the date of first surgery to that of death from any cause or to the date last known alive. Four patients of our cohort did not have surgery and were included from the date of first diagnosis of PPGL. The date of death and the cause of death (ICD-code) were obtained from the death registry of Statistik Austria, the national death registry of Austria, from hospital charts or from relatives of the patients. The latter two were contributing to less than 5% of all identified deaths as the only source. Data of the Austrian death registry are current until December 31, 2022. An additional four deceased patients were identified between January 1, 2023 and December 31, 2023 (the end of the study period) through personal contact with family members. Given that this number was not lower than the mean annual death rate in our cohort, we have chosen to include these four patients in the analyses. Patients with PPGL are at risk of MACE and death from acute cardiovascular events (6–10). DSS as defined by the number of years from first surgery to the disease-specific death or the date last known alive, was therefore analyzed using two definitions: first, death due to PPGL-related oncological causes (DSS1), second, DSS1 plus death due to CVD (DSS2).

The length of RFS was determined as the number of years from first surgery to the date of first occurrence of metastatic or nonmetastatic recurrence of PPGL or to the date last seen recurrence-free. The post-recurrence survival (PRS) was calculated as the difference of OAS minus RFS in relapsing patients. Recurrence was diagnosed by histopathological examination (either at autopsy or of a biopsy or surgical specimen) or by F-DOPA based PET-CT scans. Elevated P-MNs or U-MNs by themselves were not considered evidence of recurrence, unless confirmed by functional imaging or histopathology. Malignant recurrence was defined by histopathological (autopsy or surgery) and F-DOPA positive evidence of PPGL in organs where chromaffin cells are not present physiologically, such as lymph nodes, bone and – as multiple lesions - the liver, nonmalignant recurrence by evidence of PPGL in the adrenals, sympathetic or parasympathetic ganglia. There was no occurrence of a single liver lesion in any patient which could have given rise to misinterpretation (23). The Kaplan-Meier method was used to estimate survival as a function of time after first diagnosis or after first recurrence and comparisons of curves were made using the log-rank test. Cox proportional hazard regression was utilized to examine potential predictor variables of OAS, DSS1, DSS2 and RFS. Additional statistical considerations are given in the **Supplementary Material**.

P-values <0.05 were considered significant. All computations were performed using GraphPad Prism version 10.1.1 for Mac, GraphPad Software, San Diego, California, USA, www.graphpad.com.

## 3 Results

### 3.1 Characteristics of patients (**Table 1**)

A total of 6887 in-patients and 755 out-patients were assessed for eligibility, of which 312 patients with a histopathological diagnosis of PPGL were identified. In a study of 165 operations of patients with PCC from France, including 23 patients with malignant tumors at the time of surgery, perioperative mortality and morbidity were significant (4 deaths, 38 other complications including 13 spleen resections and hematomas), but mortality was not related to PCC (28). In our hospital, two patients with PPGL died within 90 days of first surgery (one due to air embolism during minimal invasive approach and one due to heart attack one month after the operation). They were excluded, as were seven additional patients (three with FU of less than 3 months and four lost to FU within 3 months after first surgery). The remaining 303 patients were followed for up to 50 years (mean ± SD 11.4 ± 9.2) years and represent the cohort of this study.

The age of our patients was 49.4 ± 16.3 years at study begin. Nonsurvivors were older than survivors (p<0.0001). There were slightly, but not significantly, more females in the entire cohort. Survivors were more often female (p=0.04).

The proportions of PCC (173 vs. 51 uPCC, 6 vs. 3 cPCC, 11 vs. 3 bPCC) and of PGL not HNPGL (21 vs. 10 aPGL, 1 vs 3 tPGL) were not different between survivors and nonsurvivors (p=0.26). There were more mPGL and more HNPGL in survivors than in nonsurvivors (6 vs zero and 13 vs 2, respectively).

Incidental discovery and diagnosis by screening were observed in 103 (34.0%) and 14 (4.6%) of the cohort, respectively, with no difference between survivors and nonsurvivors. Adrenergic symptoms with or without hypertension were leading to the diagnosis of PPGL in 96 patients (31.7%), more often in survivors than in nonsurvivors (p=0.04). Evaluation for uncontrolled or secondary hypertension gave rise to the diagnosis of PPGL in 22 (7.3%) and 15 (5.0%) of patients and no difference was observed between survivors and nonsurvivors. MACE lead to the diagnosis of PPGL in 18 patients (5.9%), more often in nonsurvivors than in survivors (p<0.0001). MACE not leading to the diagnosis of PPGL jeopardized the life of 26 patients (8.6%), not different in survivors versus nonsurvivors. All MACE together occurred in 44 patients (14.5%), more often in nonsurvivors than in survivors (p<0.0001). Details of patients with incidentally detected PPGL and those with MACE are given in **Supplementary Tables 1**, **2**.

Comorbidities were detected in 152 patients (50.2%), no comorbid disease and RF of CVD in 44 (14.5%) and 107 (35.3%) patients, respectively. CVD, patients with malignancy dominated disorders and those with both CVD and malignant disease were prevalent in 81 (26.7%), 52 (17.2%) and 19 (6.3%) of the cohort. Patients without comorbidities and CV RF (but no established CVD) were more common in survivors than in nonsurvivors (p<0.0001 for both comparisons), whereas CVD was more frequent in nonsurvivors than in survivors (p=0.02). When the prevalence of CVD was compared to the other comorbidities combined, that difference became nonsignificant. Details of comorbidities before first diagnosis of the total cohort are given in **Supplementary Table 3**.

Details of biochemical test results and imaging data are given in the **Supplementary Material** (**Supplementary Text** and **Supplementary Table 4**).

Family history was positive in 48 patients (15.8%) and negative in 228 (75.2%). No difference was observed between survivors and nonsurvivors regarding both the prevalence of positive and negative family history. The number of patients with unknown, as compared to positive and negative family history was smaller in survivors than in nonsurvivors (<0.0001 for both comparisons). Pathogenic variants in one of the driver genes were detected in 47 of the 48 patients (97.9%) with a positive family history and in 36 (15.8%) of those with negative family history (**Supplementary Table 4**).

Germline genetic analyses were available for 279 patients (92.1%), positive in 96 (31.4%) and negative in 184 (60.7%). Four additional patients were diagnosed with NF1 due to clinical criteria. There was no difference of positive or negative findings between survivors and nonsurvivors. 17 nonsurvivors (23.6%) and 7 survivors (3%) had missing genetic information (p<0.0001), excluding the four with clinically diagnosed NF1. Pathogenic variants of cluster 1A, 1B and 2 genes were detected in 29, 14 and 53 patients with positive genetic results (30.2%, 14.6% and 55.2%), respectively (**Table 1** and **Supplementary Table 7**).

Details of radiological imaging results, surgical approaches, experience of surgeons, duration of surgery, PASS and GAPP-score, decades of surgery and years of last FU of survivors or death of nonsurvivors, as appropriate, are given in the **Supplementary Material** (**Supplementary Text** and **Supplementary Table 4**).

Details of metastatic and nonmetastatic recurrences are given in the **Supplementary Material**.

### 3.2 Survival

#### 3.2.1 Overall survival, disease-specific survival 1 and 2

A total of 72 patients (23.8%) died during the study period. The duration of FU was not different (p=n.s.) between survivors and nonsurvivors (11.4 ± 9.4 vs. 11.1 ± 8.7 years, respectively) nor between patients with PPGL-associated oncological death (n=15), death from cardiovascular disease (n=29) or from malignant and other causes (n=28), respectively (6.9 ± 5.6 years vs. 11.7 ± 10.3 years vs. 12.7 ± 7.8 years). More survivors than nonsurvivors were seen in the 2020s (p<0.0001). Most nonsurvivors died in the 2010s.

**Figure 2:**
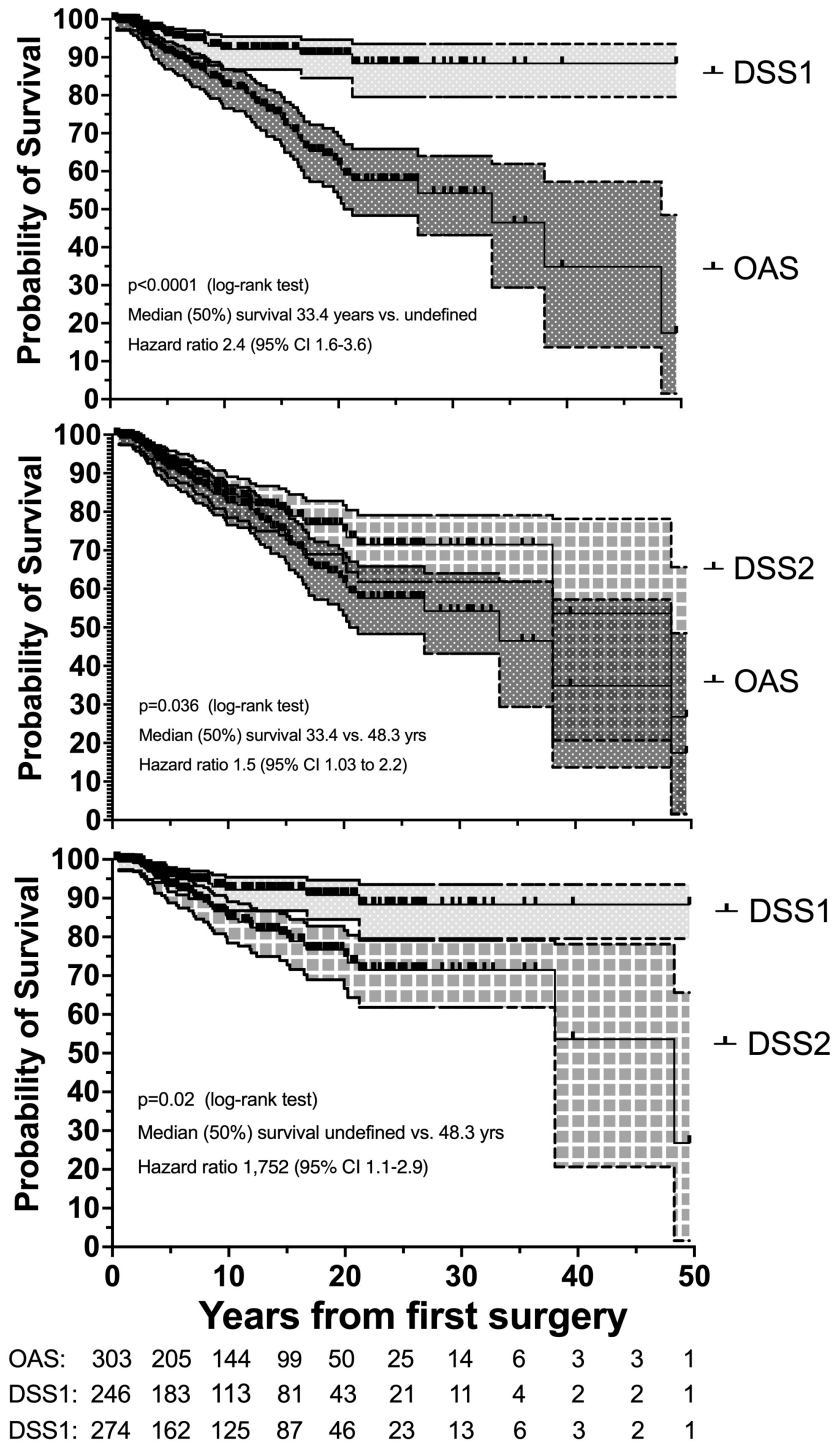
Disease-specific survival 1 (DSS1 with 95% CI) is better than DSS1 plus cardiovascular survival (DSS2 with 95% CI), and both are better than overall survival (OAS with 95% CI) in 303 patients with PPGL with a follow-up of up to 50 years. Number (#) at risk denote the number of patients at risk to be censored due to death or loss to FU at the start of the study and after each of the given 5-year intervals.

**Figure 3:**
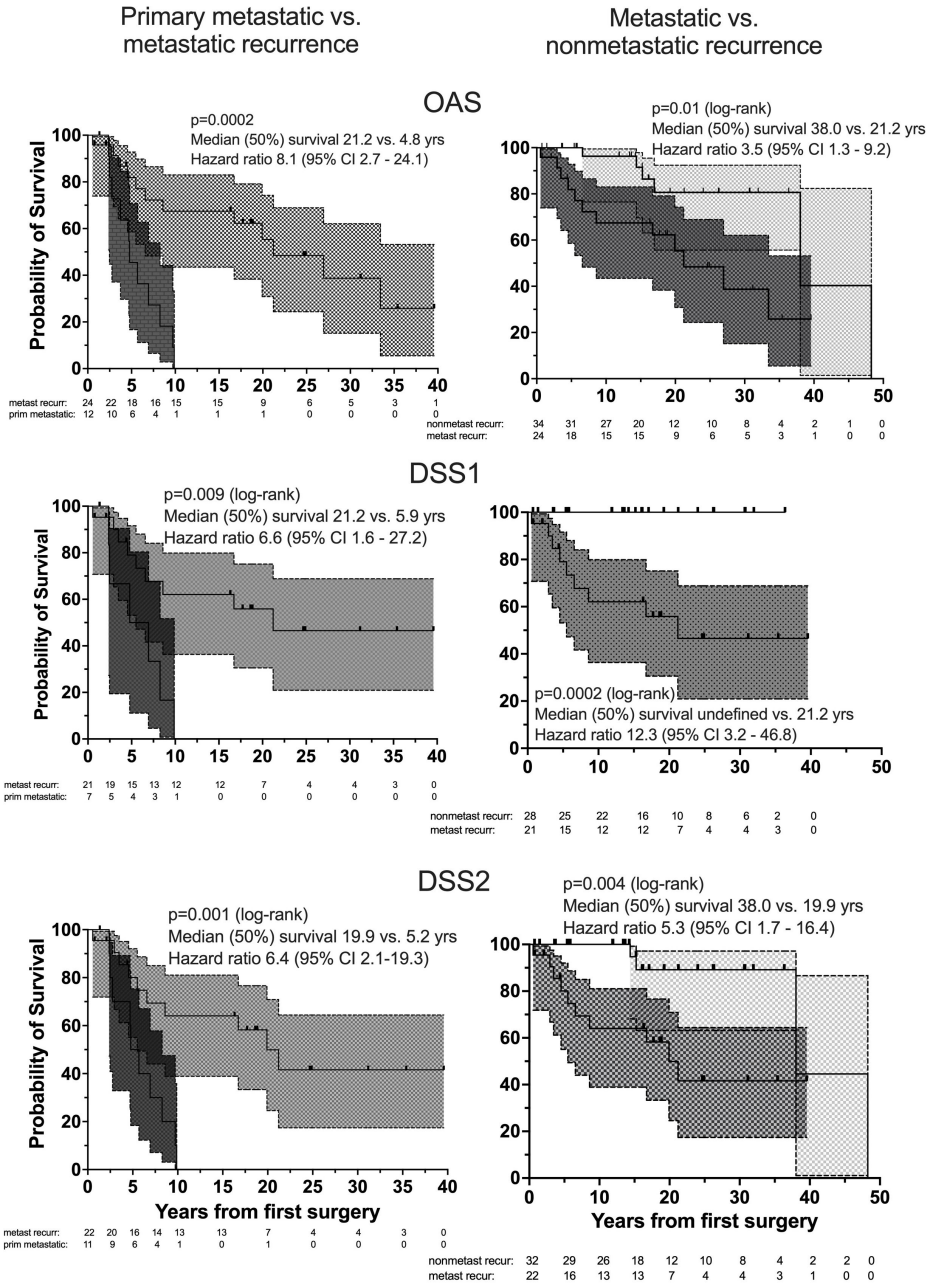
Overall survival, OAS (top), DSS1 (middle) and DSS2 (bottom) of patients with primary metastatic (dark gray), metastatic recurrent (medium gray) and nonmetastatic recurrent (light gray) PPGL by Kaplan Meier curves with 95% CI bands (left: comparison between patients with primary metastatic disease and metastatic recurrence, right: between metastatic and nonmetastatic recurrent PPGL). Number (#) at risk denote the number of patients at risk to be censored due to death or loss to FU at the start of the study and after each of the given 5-year intervals.

**Figure 4:**
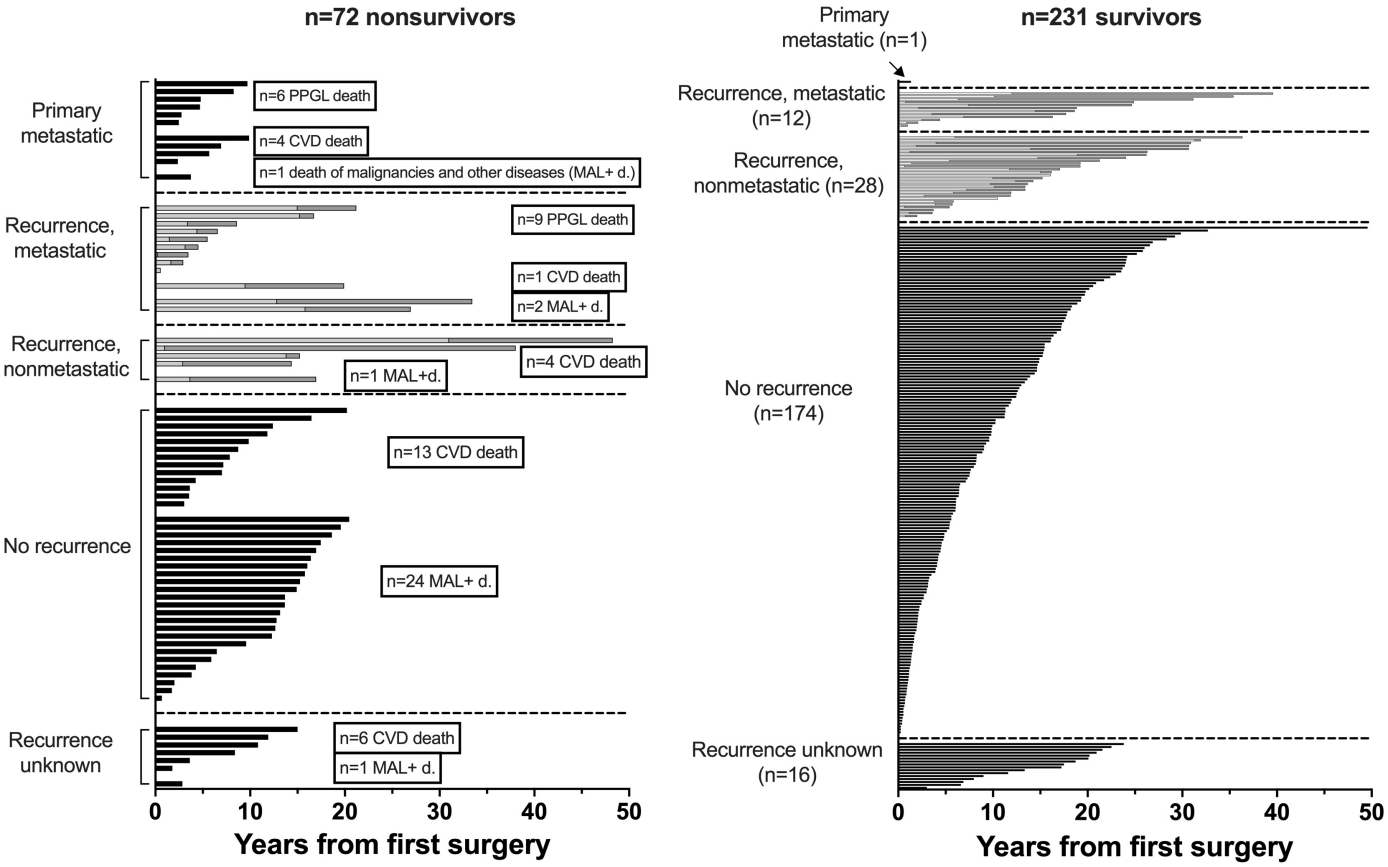
Waterfall plot of overall survival (OAS, black bars), recurrence free survival (RFS, bright bars) and post recurrence survival (PRS, grey bars) over a follow-up of 50 years of 303 patients with PPGL (RFS+PRS=OAS in recurrent disease).

Nonsurvivors died with comparable relative frequency (p=0.40) from PPGL, CVD or malignant disease in the 1990s, 2000s, 2010s and 2020s (**Supplementary Table 11**). Patients with primary metastatic disease had the worst prognosis: 11 of 12 died after a FU of 5.2 ± 2.9 (median 4.8) years. DSS1 was better than DSS2 (p=0.02) and both better than OAS (p<0.0001 and p=0.036, for the comparison DSS1 vs. OAS and DSS2 vs. OAS, respectively), when the total cohort was considered (**Figure 2**). At 50 years of FU, the DSS1 was still 88% (95% CI 80-93). The 5-, 10-, 20- and 30-year rates of OAS, DSS1 and DSS2 are summarized in **Table 2**. The Kaplan Meier product limit estimates of the entire cohort are shown in **Figure 2**, those of patients with primary metastatic disease vs. metastatic recurrence and of metastatic vs. nonmetastatic recurrences in **Figure 3**. A Waterfall plot displaying the length of OAS, RFS and PRS of all 303 patients divided by primary metastatic disease, metastatic and nonmetastatic recurrence, no recurrences as well as unknown recurrence is shown in **Figure 4**. Details of causes of death of the 72 nonsurvivors are given in **Supplementary Table 8**.

**Table 2:** Summary of the 5-, 10-, 20- and 30-year survival rates for death of all causes (OAS), PPGL-related death (DSS1) and death due to DSS1 and cardiovascular diseases (CVD) combined (DSS2), including 95% confidence intervals (95% CI) of the total cohort, as well as of patients with metastatic and with nonmetastatic recurrences.

Survival	Total cohort (n=303)		Metast rec (n=24)		Nonmetast rec (n=33)	
	% (95% CI)	# at risk	% (95% CI)	# at risk	% (95% CI)	# at risk
5-year OAS	91 (87-94)	205	82 (58-93)	31	100 (100-100)	18
5-year DSS1	96 (94-98)	162	79 (53-92)	15	100 (100-100)	25
5-year DSS2	93 (89-96)	183	80 (55-92)	16	100 (100-100)	29
10-year OAS	82 (77-87)	144	67 (43-83)	15	96 (76-99)	27
10-year DSS1	92 (87-95)	113	62 (37-80)	12	100 (100-100)	22
10-year DSS2	85 (78-89)	125	64 (39-81)	13	100 (100-100)	26
20-year OAS	63 (54-70)	50	55 (34-74)	9	81 (56-92)	12
20-year DSS1	91 (85-95)	43	56 (31-75)	7	100 (100-100)	10
20-year DSS2	75 (69-82)	46	50 (25-71)	7	89 (64-97)	12
30-year OAS	54 (43-64)	14	39 (15-62)	5	80 (56-92)	8
30-year DSS1	88 (80-93)	11	46 (21-69)	4	100 (100-100)	6
30-year DSS2	71 (62-79)	13	42 (18-65)	4	89 (64-97)	8

Number (#) at risk denote the number of patients at risk to be censored due to death or loss to FU after the 5-, 10-, 20- and 30-year time points.

Details as to recurrence free survival (RFS) and post recurrence survival (PRS) are given in the **Supplementary Material**.

### 3.3 Prognostic factors for survival

After fitting the Cox proportional hazard model, the key predictor parameters of shorter OAS probability included primary metastatic disease (HR 11.1, 95% CI 4.1-27.9), MACE (HR 2.7, 95% CI 1.2-6.1), comorbid malignancies irrespective of potential additional CVD (HR 2.5, 95% CI 1.3-4.9, male sex (HR 2.0, 95% CI 1.1-3.6) and higher age (HR 1.05, 95% CI 1.03-1.07). The key predictor parameters of shorter DSS2 were primary metastatic disease (HR 23.3, 95% CI 8.1-62.5), male sex (HR 2.6, 95% CI 1.2-5.6), MACE (HR 4.5, 95% CI 1.7-12.2) and higher age (HR 1.06, 95% CI 1.03-1.09). The small number of tumor-related deaths (15 patients) and the overwhelming prognostic influence of primary metastatic disease on DSS1 (HR 41.8, 95% CI 13.2-129) precluded the evaluation of potential additional risk factors (**Table 3**).

**Table 3:** Predictive factors for overall survival (OAS), disease-specific survival 1 (DSS 1), disease-specific survival 2 (DSS 2) and recurrence-free survival (RFS, all recurrences) for 303 patients with PPGL by Cox regression.

	Reference	OAS	p-value	DSS 1	p-value	DSS 2	p-value	RFS	p-value
		HR (95% CI)		HR (95% CI)		HR (95% CI)		HR (95% CI)	
Age	per year	1.05 (1.03-1.07)	<0.0001			1.06 (1.03-1.09)	<0.0001	0.96 (0.93-0.99)	0.0006
Sex	female	2.0 (1.1-3.6)	0.02			2.6 (1.2-5.6)	0.01		
Location	PCC								
	*PGL*			2.5 (0.5-9.0)**	0.20				
	* HNPGL*			0.2 (0.02-1.3)**	0.13				
Comorbidity	No disease								
	*CVD*							4.0 (1.7-9.2)	0.001
	*Mal*	2.5 (1.3-4.9)	0.005					1.3 (0.7-2.7)	0.41
	*Mal+CVD*							0.7 (0.1.2.6)	0.65
Symptoms	Typical								
	*MACE*	2.7 (1.2-6.1)	0.01	3.6 (0.73-14.6)**	0.08	4.5 (1.7-12.2)	0.002		
	*Oligosympt*	1.6 (0.8-3.2)	0.19	1.02 (0.2-4.1)**	0.98	2.0 (0.8-5.1)	0.14		
Secretory	yes								
Tumor size	per cm								
Meta at first diagnosis	no	11.1 (4.1-27.0)	<0.0001	41.8 (13.2-129)*	<0.0001	23.3 (8.1-62.5)	<0.0001		
Metast rec	no								
Genetics	neg							3.1 (1.5-6.8)	0.002
Genetic cluster	neg								
	*Cluster 1A*							11.1 (4.7-27.5)§	<0.0001
	*Cluster 1B*							0.9 (0.2-3.2)§	0.87
	*Cluster 2*							1.7 (0.7-4.1)§	0.26

§ HR for RFS of genetics (all positive vs. all negative findings) and of the three clusters are from two different models, keeping the other parameters constant. These two models included 9.6 and 8.7 parameters per event, respectively. When all positive genetic findings were replaced by the three clusters (RFS), the significance of the other parameters remained unchanged. *Given the low number of events (n=15 deaths from PPGL), the predictive model for DSS1 precluded the analysis of more than one factor. ** Exchanging symptoms before diagnosis (or other dependent variables) for primary metastatic disease led to statistical overfitting for DSS1 and was not better than the simpler model containing no covariates (the crossed-out HR and p-values for location and symptoms before diagnosis are for illustrative purposes only).

When only considering patients at risk for metastasis after first surgery (e.g. excluding those with metastatic disease at first diagnosis), independent prognostic factors changed only slightly. Comorbid malignant disease (HR 4.6, 95% CI 2.0-11.0) instead of malignant with or without CVD was predictive of shorter OAS and metastatic recurrence (HR 2.9, 95% CI 1.02-7.8) of shorter DSS2. All nine deaths due to PPGL-related causes could be predicted by metastatic recurrence, precluding the evaluation of any other variable (**Supplementary Table 12**).

Predictive factors for recurrence-free survival are given in the **Supplementary Material** (**Supplementary Text** and **Supplementary Table 13**).

## 4 Discussion

Our single-center cohort of 303 patients with newly diagnosed PPGL was followed for 11.4 ± 9.2 (mean ± SD, range 0.3-50) years. We present the first study, that meets the FU duration of at least 10 years recommended by the European Society of Endocrinology for patients with PPGL (29). To the best of our knowledge, this is the first single-center study assessing comorbidities, detailed symptoms/presentations leading to the diagnosis of PPGL and other characteristics in a large unselected cohort of patients with PPGL and using these parameters for analysis of overall and disease specific survival, as well as recurrence-free survival during a sufficiently long FU.

### 4.1 Clinical presentation and prognostic implications

In our cohort, there were 111 (36.6%) patients with incidentally discovered PPGL, 14 (4.6%) patients diagnosed due to screening and 165 (54.5%) patients detected due to clinical suspicion. Those identified by chance were older and had genetic abnormalities less frequently as compared to PPGL detected due to either clinical suspicion or screening, which is in line with other authors (3). PPGL have recently reported to be most frequently discovered incidentally (4, 30, 31), with up to 69% of 132 patients with PCC in one series from Great Britain. Others, on the other hand, have reported lower rates of incidentally discovered patients (32–34). Our patients were most commonly diagnosed due to clinical suspicion. Tumor size did not differ irrespective of the mode of discovery which is in contrast to others reporting that incidentally discovered PPGL were smaller than those detected due to clinical suspicion, but larger than tumors identified due to screening (30). In our study, larger tumor size was predictive of metastatic recurrence and DSS1, while smaller tumor size was predictive of nonmetastatic recurrence. Larger tumor size has been reported to be predictive of recurrent PPGL by some (35), but not all (18, 36), of shorter OAS by some (37–39), but not all (16, 18, 35, 36, 40, 41) and of shorter DSS by some (12, 38), but not all (14, 16, 41) authors. The heterogenous design as to patient selection, some studies pulling from national or regional cancer databases, others being single center or multicenter studies, with variable proportions of patients with malignant PPGL either at diagnosis or during FU, differences in the frequency of genetic testing and of positive genetic results or distinct pathogenic gene variants and the highly variable duration of FU limit the comparability among different studies. It has been acknowledged that up to 40% of patients with incidentally detected PPGL in fact suffered from adrenergic symptoms prior to diagnosis, symptoms that had apparently gone unnoticed by patients and/or their physicians (30, 42). The proportion of patients with PPGL suffering from MACE prior to diagnosis or the impact of different modes of clinical presentations on DSS have not yet been studied. In our study, 36 of the 103 patients (35.0%) with incidentally discovered PPGL were in fact highly symptomatic, 14 with ≥2 typical adrenergic symptoms and 22 with MACE a median time of 6 (range 0.5-60) months prior to first surgery. A total of 44 patients (14.5% of the study cohort) suffered from life-threatening MACE prior to the discovery of the tumors. This incidence is in line with others reporting a frequency of 18-19% life-threatening cardiovascular complications in patients with PPGL (7, 43). In 26 of these 44 patients (59.1%), the MACE did not lead to clinical suspicion of PPGL (the diagnosis established by chance in 22 and due to diagnostic work-up for uncontrolled hypertension in 4 patients), not more frequently in nonsurvivors than in survivors. Four of these 26 patients survived a second MACE 12, 18, 26 and 35 months after the first, respectively (all prior to first diagnosis of PPGL). In 18 patients, the MACE was the key factor for the discovery of PPGL, more frequently identified in nonsurvivors than in survivors. Two of the 44 MACE (two transient ischemic attacks during hypertensive crisis) occurred during core needle biopsy of the adrenal tumor prior to biochemical assessment. A recent systematic review of 56 studies including a total of 86 patients (34% with metastatic disease) with a history of core needle biopsy (CNB) reported a 23.1% incidence of complications. No CNB related death was described, but complications requiring hospitalization or intervention occurred in 4 of 27 patients (two AMI, one Takotsubo syndrome, one temporal duodenal obstruction caused by hematoma) and CNB related catecholamine symptoms including hypertensive crisis in 8 of 25 patients (44). In our cohort, MACE was the risk factor with the second largest hazard ratio for death due to all causes (HR 2.7, 95% CI 1.2-6.1) as well as due to PPGL and CVD together (HR 4.5, 95% CI 1.7-12.2). Incidental diagnosis did not confer any prediction of survival. Of note, adrenergic symptoms that to physicians may be most suggestive of PPGL was chosen as reference in the Cox regression (**Table 3**). Data regarding missed clinical clues and a delayed diagnosis of PPGL have been published previously (45), but the prognostic significance as to survival of both has not been evaluated. In a population-based study from Denmark, median duration of symptoms prior to the diagnosis of PPGL of a cohort of 192 patients was 1.7 years, with 26.4% having symptoms for ≥5 years (46). In our study, MACE not leading to the diagnosis of PPGL occurred well within 5 years before first diagnosis. Thus, the high catecholamine serum concentrations due to undiagnosed secretory PPGL over a period of months or even years may have been at least contributory to the MACE. Catecholamine induced damage to the heart and vessels is well known in PPGL (6, 47) and is, in part, reversible after adrenalectomy for PCC (48, 49). Left ventricular ejection fraction improves after surgical therapy, but systolic and diastolic myocardial strain impairment, as well as focal and diffuse myocardial necrosis identifiable as cardiac MRI abnormalities persist beyond those seen in hypertensive patients alone (50).

### 4.2 Survival

Overall mortality of our cohort was 23.8% (72 of 303 patients). This is comparable to the mortality of adrenal adenomas in a population-based setting (51). Although the proportion of patients with CVD in our cohort (26.7%) was similar to that at time zero of those of the adrenal adenoma study (all different CV entities together 16-32%), patients with adrenal adenomas were older, individuals <20 years excluded and FU duration was shorter than in our cohort. In addition, the patterns of survival curves differ. The linear increase of mortality of adrenal adenomas contrasts with the comparatively steeper curves of our patients with PPGL. In our study, the death rate due to all causes was higher in first 20 years of FU, in the first 5-10 years of patients with metastatic recurrence and highest in the first 5 years in patients with primary metastatic disease (**Figure 3**). In our cohort, OAS was independently predicted by age, sex, malignant comorbidities, MACE and primary metastatic disease. When assessing survival of patients with PPGL, most studies refer to overall survival. However, 28 of 72 nonsurvivors (38.9%) of our study died of causes unrelated to PPGL and in an additional 29 patients (40.3%) the cause of death was cardiovascular, leaving a tumor-related (oncological) mortality of 5.0% of the total cohort.

There is a great heterogeneity in disease-specific outcome studies. Between 1997 and 2023, we have identified 17 studies (12–17, 32, 33, 52–60) reporting number and proportion of patients with disease-specific death (DSD), seven that included 5- and 10-year DSS (12, 14, 17, 38, 56, 59, 60) and three with analyses of risk factors of DSS by Cox regression analysis (12, 14, 16). In the four studies including patients with malignant PPGL only, DSD was uniformly defined as tumor-associated and ranged from 26.8% to 46.2% (12, 13, 16, 56). The participants in a study from the NIH were highly selected, included 27 (20.5%) children <19 years and 73 of 132 patients had pathogenic variants of the SDHB gene (56). Two studies including patients with bilateral PCC (53, 54) and one with locally advanced PPGL (15) described ≤5 patients with DSD, corresponding to a DSD rate of 4.8% to 5.5% over a median FU period of 4.5 to 8.5 years. Another two studies of PCC only and of secreting PPGL reported ≤6 DSD, corresponding to a DSD rate of 2.6% to 5.1% (17, 32, 59). These findings are in line with the results of our DSD rate (15 PPGL-related oncological deaths, 5.0%). The 5-year and 10-year DSS (including 95% CI) in studies of patients with exclusively malignant PPGL ranged from 62% to 96.2% (12, 13, 38, 56) and from 63.5 to 86.4% (12, 38, 56) respectively. In studies, that also included patients without metastatic disease at the beginning, the 5- and 10-year DSS (including 95% CI) ranged from 92.2 to 100% and 86.7 to 100%, respectively (17, 59, 60). The DSS of our study (**Table 3**) are in line with these results. RF for DSD in studies investigating only patients with metastatic PPGL were higher age (12, 16), larger tumor size (12), primary metastatic (12) or all distant metastatic recurrent (16) disease and no surgical therapy (16). In an analysis of the SEER database, Mei L et al. reported hazard ratios of 14 independent variables and of six significant risk factors for DSS (higher age, male sex, a second primary malignancy, aortic/carotid body PGL, distant metastasis and higher TNM stage), but did not describe the number of events nor whether the analysis was univariate or multivariate (41). A systematic review and meta-analysis of patients with metastatic PPGL of 20 retrospective noncomparative studies identified 1338 patients with a mean FU of 6.3 ± 3.2 years. The 5-year (7 studies, n=738 patients) and 10-year (2 studies, n=55 patients) overall mortality rates were 37% (95% CI, 24%-51%) and 29% (95% CI, 17-42%). Higher mortality was associated with male sex and synchronous metastases (61).

There is only one study including death due to CVD in the DSS and reporting predictive factors for that definition of DSS in a cohort of patients with and without metastatic PPGL (14). This study comprised of 639 patients (407 PCC, 175 PGL not HNPGL and 57 HNPGL) from 6 tertiary European centers and from one quaternary referral center in the USA. DSD was defined differently to our study, however, including not only death due to events, that could have been associated with previous long-term or current catecholamine excess (e.g. CV manifestations), but also death caused by peri- or postsurgical complications, metastatic disease or treatment complications. The respective contributions of these causes of death to the 5- and 10-year DSS of 86.1% and 59.8% of 209 (190 PPGL not HNPGL, 19 HNPGL) patients with metastatic (35.9% of the study cohort) and 98.6% and 97.2% of 430 (392 PPGL not HNPGL, 38 HNPGL) patients without metastatic disease were not given. 15% of the cohort presented with a history of recurrent disease at the beginning of the study, more patients (100 of 549 PPGL not HNPGL, 6 of 57 HNPGL, together 16.5%) than of our cohort harbored pathogenic mutations in the SDHB gene, median FU was shorter and the definition of DSD differed from those of DSS1 (PPGL-related) and of DSS2 (PPGL- and CVD-related) of our study. The results are thus not entirely comparable to our study. Still, the long-term DSS after 5, 10 or 20 years were comparable.

Our study has limitations, including the lack of determination of dopamine metabolites, that have been shown to be predictive of DSS in some (14), but not all (12) studies. Elevated dopamine concentrations in urine provide a poor marker of a dopaminergic phenotype (14). However, less than 5% of our patients presented with urinary dopamine concentrations greater than the upper limit of the normal reference range and the biochemical phenotype failed to show a significant association with either recurrence or survival in our study. In addition, the number of events remained rather small, preventing the analysis of more than a few potential risk factors. This is an inherent dilemma of a rare disease, which may be overcome with a multicenter design, yet at the expense of generalizability to clinical practice. Also, we did not assess therapeutic measures other than surgical interventions. However, there is no cure for metastatic PPGL and surgery remains the only effective therapy until today (62). Despite these limitations, our study has unparalleled strengths. We were able to retrieve detailed clinical, biochemical and genetic data and present largely unbiased detailed outcome of the largest single-center cohort reported to date. Our study presents outcome data over the longest follow-up period of patients with PPGL, a study strength that minimized misclassification of patients with metastatic potential among those without evidence of metastases. Of note, two thirds of all patients repeatedly presented to one of us (W.R.) during the last 3 ½ years of the study. We were thus able to prospectively assess comprehensive clinical and up-to-date germline genetic data. Our results are representative of a tertiary referral hospital serving a population of approximately two million.

## 5 Conclusions

In summary, this study identified major adverse cardiovascular events such as acute myocardial infarctions or stroke prior to diagnosis, occurring in 44 (14.5%) of our patients, as predictors of both shorter overall and disease-specific survival, defined as death due to PPGL-related causes and cardiovascular disease (CVD) for the first time. Primary metastatic disease was the predominant predictive factor for death due to PPGL-related causes, metastatic recurrence predictive of shorter survival due to oncological causes in patients with nonmetastatic disease after the first operation. Higher age and male sex remained independent predictors of death due to all causes and due to PPGL plus CVD. Shorter recurrence free survival was predicted by lower age, CVD prior to first surgery and cluster 1 pathogenic germline variants. There was no disease-specific death in any patient with nonmetastatic recurrences.

## Data Availability

The original contributions presented in the study are included in the article/**Supplementary Material**. Further inquiries can be directed to the corresponding author.

## Author’s note

Presented in part at the 2022 Annual Meeting of the Endocrine Society: Raphael Schendl, Christoph Krall, Wolfgang Raber, OR12-5 | LBSAT57 Long-term Follow-Up of Pheochromocytoma/Paraganglioma (PPGL) after first diagnosis: a retrospective single-center study of 173 patients, Journal of the Endocrine Society, Volume 6, Issue Supplement_1, November-December 2022, Page A85, https://doi.org/10.1210/jendso/bvac150.175.
