# Supplementary material for: Metastatic disease and major adverse cardiovascular events preceding diagnosis are the main determinants of disease-specific survival of pheochromocytoma/paraganglioma: long-term follow-up of 303 patients
Source: Front Endocrinol (Lausanne). 2024 Aug 21;15:1419028. doi: 10.3389/fendo.2024.1419028 (PMC11371702; doi:10.3389/fendo.2024.1419028)
Supplement: Supplementary file 1 [file DataSheet1.pdf]

Supplementary Material

2 Materials and Methods

2.1 Patients

The database for in-patients was searched for Codes of the International Classification of Disease Version 10 and 9 (ICD-10 and ICD-9) E.27.5, 255.6 (adrenomedullary hyperfunction), C74.1 (malignant neoplasm, adrenal medulla), D35.5, C75.4, D44.6 (carotid body neoplasm, benign, malignant and of uncertain or unknown behavior), D35.6, C75.5, D44.7 (aortic body and other PGLs, benign, malignant and of uncertain or unknown behavior), D44.8, 258.0 (neoplasm of uncertain or unknown behavior, pluriglandular involvement including multiple endocrine neoplasia 2A, MEN-2A), Q85.8 (von Hippel Lindau- disease, VHL), Q85.0 (neurofibromatosis type 1, nonmalignant, NF1), I15.2 (hypertension secondary to endocrine disorders), D35.0, D44.1 (adrenal gland, benign neoplasm and neoplasm of uncertain or unknown behavior). The database for patients of our endocrinological out-patients unit was searched for the following terms: "PHEO", "PCC operated", "PCC suspected", "Plasma-Metanephrines (P-MNs) abnormal", "24h Urinary Metanephrines (U-MNs) abnormal", "PGL", "MEN-2A", "NF", "VHL", "familial disease".

2.2 Methods

2.2.2 Biochemical evaluation and imaging

2.2.2.1 Biochemical evaluation

Biochemical evaluation for the detection of PPGL has changed over the years. In the 1980s, the determination of plasma catecholamines (P-CATs) was the only available method, largely replaced by 24h-urinary catecholamines (U-CATs) in the 1990s. It was not before the beginning of the new millennium,

that 24-hour urinary metanephrines (U-MNs) and plasma free metanephrines (P-MNs) became the gold-standard (1). Most patients had more than one laboratory test on multiple occasions before diagnosis of PPGL. For each method, only the highest reported peroperative concentrations were considered.

Between March 1999 and August 2002, respectively, and March 2023 (date of inclusion of the last patient to the study), a total of 159 (52.5%) and 132 (43.6%) patients have preoperatively been assessed with U-MNs and P-MNs, respectively and both synchronously in 112 (37.0%) patients (Suppl. Table 4). From July 1990 until today, preoperative biochemical testing was performed by U-CATs in 226 (74.6%) patients and synchronously with U-MNs in 117 (38.6%). Before 1990, nine patients (3.0%) had determinations of P-CATs (Suppl. Table 4). Alltogether, preoperative biochemical test results were available for 259 (85.5%) patients.

Concentrations of test parameters are given as multiple of the upper limit of normal (ULN) of the reference intervals to allow for easy comparisons between methods.

2.2.2.2 Imaging

All patients had preoperative diagnostic imaging: 267 (88.1%) by either CT or MRI, 201 (66.3%) by [18]Fluoro-dihydrophenylalanine (F-DOPA) PET-CT, [18]Fluoro-deoxyglucose (FDG) PET-CT or metaiodobenzylguanidine (MIBG) scintigraphy (Suppl. Table 4). Most patients had a combination of more than one imaging method including more than one examination by conventional methods.

2.2.4 Family history and germline genetic analysis

Approximately 40% of PPGL patients harbor a germline mutation in one of the about 20 susceptibility genes known so far (2). The knowledge of pathogenic gene variants has been evolving. In 1990, the NF1 gene was cloned (3), germline mutations of the RET proto-oncogene in MEN-2A (4) and of the tumor suppressor gene *VHL* were identified in 1993 (5). In 2000, gene mutations in the *SDHD* and the *SDHC* genes, and in 2001, pathogenic variants in the *SDHB* gene subunit were found to be causative to familial PPGL (6–8). Thus, patients in the 1990s, 2000s and early 2010s were tested for pathogenic

variants in only a few genes such as *RET*, *SDHB-D* and *VHL*. Later, more and more driver genes were identified (2). The methodology as performed in our patients during these years has been described previously (9–12). Since the mid-2010s, next generation sequencing (NGS) has largely replaced former technologies and has become the method of choice at our institution, since 2020 supplemented by a neuroendocrine gene panel, both outlined below.

2.2.4.2.1 Exome sequencing and bioinformatics analysis (NGS).

Genomic DNA was extracted from peripheral blood according to standard protocols. For about 40% of the 153 patient samples of our cohort, multigene analysis was enriched for 6713 genes associated with Mendelian disorders by means of the TrueSight One Expanded Sequencing Kit (Illumina, San Diego, California, USA). For the remainder, enrichment for whole exome analysis was perforrmed with either the TrueSeq Exome Kit (Illumina), or the Twist Comprehensive Exome Panel (TWIST Bioscience, San Francisco, California, USA). DNA fragments were paired-end sequenced on an Illumina NextSeq500 for multigene analysis or an Illumina NextSeq2000 system for whole exome analysis. The obtained sequencing reads were alligned to the NCBI (National Center for Biotechnology Information, an US government agency providing access to biomedical and genomic information for science and health) human genome assembly (hg19) using the Burrows Wheeler Alignment Tool. Variant calling (HaplotypeCaller) was performed according to GATK (Genome Analysis Toolkit) best practice guidelines for calling single-nucleotide variants, insertions and deletions (13). The evaluation of the called variants was performed using VarSeq software of Golden Helix (Bozeman, Montana, USA). The exome wide read coverage was 75, that of the multigene panel 110. Variants were filtered based on minor alle frequency (MAF) using in-house database including data from >1000 whole exomes and published disease-causing variants. Heterozygous and homozygous variants present in more than five and more than three cases, respectively, were extracted, followed by filtering based on the MAF listed in the Genome Aggregation database (14), MAF ≤ 0.5. The remaining variants were searched for genes associated with the HPO (Human Phenotype Ontology) terms “pheochromocytoma, paraganglioma, neuroendocrine neoplasm” of the Omim (Online Mendelian Inheritance in Man) database, an online catalogue of human genes and genetic disorders (15), with predefined criteria: read of >9, current allele frequency of >0.35 and Phred scale base quality score >99. In a second step, the cutoff for the variant allele frequency was lowered to 0.19 to allow the detection of possible mosaicism.

Six prediction tools for independent assessment of the pathogenicity of filtered missense variants were used routinely: SIFT, Polyphen2 HVAR, MutationTaster, MutationsAssessor, FATHMM and FATHMM MKL Coding. Variants with at least four predictions as damaging, pathogenic, or likely pathogenic were selected. Loss of function variants were independently considered for further analysis. All variants were assessed as to their pathogenicity by the ClinVar database and the Leiden Open Variation Database, LOVD (16,17). Reported variants were classified based on the American College of Medical Genetics and Genomics (ACMG) guidelines (18). In addition, a copy number variation (CNV) analysis was performed for all analyzed samples comparing the calculated coverage of each sequenced sample to the already existing coverage database obtained from BAM (binary alignment map) files for all previously analyzed in-house samples. This analysis was performed by a supported module of VarSeq within the VarSeq software of Golden Helix.

2.2.4.2.2 Neuroendocrine gene panel

Genomic DNA was extracted from peripheral blood according to standard protocols. Enrichment of target genes was performed by a Custom Capture gene panel (Twist Bioscience, San Francisco, USA), followed by massively parallel sequencing on the NextSeq550 system of Illumina. Automatic alignment of sequences was against the human reference sequence GRCh38, the genome assembly Genome Reference Consortium Human Build 38 (hg38) of NCBI (19). Variant classification was according to the ACMG guidelines,and the Association for Clinical Genomic Science (ACSG) best practice guidelines for variant classification in rare disease (18,20). The criteria for analysis were as follows: determination of target regions with a minimum 20-fold coverage, only variants with a maximum frequency in the population (MAF gnomAD and ExAC database) <1% within coding regions and flanking intron regions (-15/+8). Causative variants as issued in the ClinVar database were considered in flanking introns up to ± 25 base pairs and within the 5´- and 3´- untranslated regions. As with the NGS analyses, variants classified as pathogenic (ACMG class 5), or likely pathogenic (ACMG class 4) were considered and analytically confirmed. Interpretation of the findings was always in the context of available clinical information at the time of the analysis. Variants of unclear significance (VUS) were issued in the annex to the findings. The following genes were covered: *CDKN1B, DLST, EPAS1, FH, GDNF, KIF1B, MAX, MDH2, MEN1, NF1, PRKAR1A, RET, SDHA, SDHAF2, SDHB, SDHC, SDHD, SLC25A11, TMEM127, VHL.*

2.3 Statistics

Univariate analysis included the following dependent variables: age at first surgery, sex, comorbidities, symptoms/clinical presentation leading to diagnosis, secretory status, tumor location, tumor size, metastatic disease at first diagnosis, recurrence of all causes, metastatic and nonmetastatic recurrence, genetic results or genetic cluster, surgical approach, experience of the surgeon and duration of surgery (Suppl. Table 6). The variables for fitting the model of the (multivariate) Cox regression were chosen according to the degree of clinical significance, as well as the consistency of results of previous studies and comprised metastatic disease at first surgery, metastatic recurrence, tumor size, location of the tumor, symptoms/clinical presentation leading to diagnosis, age, sex, comorbidities, genetic results and secretory status. In evaluating the models, due care was taken to keep the ratio of the number of events per parameter close to ten (21). The event variables indicated whether patients had died of any cause (OAS), of PPGL (DSS1) or of PPGL and CVD together (DSS2), or whether they were diagnosed with recurrence (metastatic and nonmetastatic) of PPGL (RFS). The time variables contained the elapsed time for each individual between first surgery (or first diagnosis in the four patients without surgery) and the time they either died, suffered a recurrence or were censored (the day of last FU in case of alive patients). RFS was equal to OAS in alive patients with no recurrence. Prognostic factors for post recurrence survival (PRS) were not studied, because PRS was heavily dependent on the length of FU and would have introduced systematic bias.

3. Results

3.1 Characteristics of patients

When compared to the oligosymptomatic and the non-MACE symptomatic group, respectively, patients with MACE were observed more often in nonsurvivors than in survivors (p=0.0005 and p=0.0001, respectively). There was no temporal trend over time (p=n.s.): MACE in the 2020s occurring in 8/43 patients (18.6%), in the 2010s in 11/98 patients (11.2%), in the 2000s in 15/93 patients (16.0%), in the 1990s in 7/54 patients (13.0%) and in the 1980s in 3/11 patients (27.3%). There was no difference in the percentage of nonsurvivors, when MACE leading or not leading to the diagnosis of PPGL were compared (11 of 18 and 10 of 26 not surviving, respectively, p=0.22). For all survival modes, the

hazard ratio (HR) of the 103 asymptomatic patients (12 of 14 diagnosed due to screening and 45 of 89 diagnosed incidentally had no symptoms) was not different (p=0.36) versus patients with adrenergic symptoms.

Biochemical results were available for 259 patients (85.5%), more often in survivors than in nonsurvivors (p<0.0001). Of note, most patients had combinations of plasma und urinary parameter determinations. Alltogether, 241 patients (93.1%) tested positive and 18 (7.1%) negative. P-MNs as compared to U-MNs and U-CATs, respectively, contributed more to positive results in survivors than in nonsurvivors (p<0.0001), both U-MNs and U-CATs more so in nonsurvivors than in survivors (p<0.0001). Over the 50 years of FU, P-MNs identified 152 patients, U-MNs 21, U-CATs 62 and P-CATs 6, contributing to 63.1%, 8.7%, 25.7% and 2.5% of positive biochemical results, respectively. U-NMN and P-NMN were the single parameters most commonly positive, followed by U-MN and P-MN, yielding 89.4%, 87.9%, 86.4% and 72.6% of correct positive results. There were no differences between survivors and nonsurvivors.

One quarter (25.4%) of the cohort had both, computed tomography (CT) and magnetic resonance imaging (MRI), 127 (41.9%) patients only CT scans and 73 (24.1%) MRI alone. For 22 patients (7.3%) conventional imaging results were unknown. There were no differences between survivors and nonsurvivors. Functional imaging was performed in 200 patients (66%). F-DOPA PET-CT was the most common method and, as compared to both FDG PET-CT and MIBG scans, was more commonly used in survivors than in nonsurvivors (p<0.001 and p=0.0008).

The open surgical approach was chosen in 114 patients (37.6%) and was more frequently used in nonsurvivors than in survivors compared to both the transperitoneal (142 patients, 46.9%) and the retroperitoneal (42 patients, 13.9%) laparoscopic approach (p<0.0001 for both comparisons). Transperitoneal laparoscopic surgery was performed more commonly in survivors than in nonsurvivors (p=0.02). The duration of surgery was longer in nonsurvivors than in survivors (p<0.0001), reflecting the greater tumor size (mean diameter 6.9 vs. 4.8cm, p<0.0001), the greater proportion of tumors >6cm (55.4% vs. 28.6%, p=0.0003) and the greater frequency of the open surgical approach in nonsurvivors. Tumor size of PPGLs discovered incidentally or due to screening procedures was not different to that of patients with symptoms (p=n.s.). Operations were performed by specialized endocrine surgeons in 234 patients (77.2%), more often in survivors than in nonsurvivors, when compared to nonspecialized surgeons (p=0.02). In the 1990s, 2000s, 2010s and 2020s, there were 33 (14.3%), 65 (28.1%), 86 (37.2%)

and 43 (18.6%) operations in surviving patients and 21 (29.2%), 28 (38.9%), 12 (16.7%) and zero in nonsurviving patients (p=0.0003 for the comparison 1990s vs. 2010s, p=0.003 for 2000s vs. 2010s, p=0.28 for 1990s vs. 2000s), respectively. PASS score <4 was observed in 137 of 171 survivors (80.1%) and in 28 of 43 (62.8%) nonsurvivors (p=0.01). On the other hand, PASS ≥4 was more common in nonsurvivors than in survivors (p=0.01). GAPP score tended to be higher in nonsurvivors. The number of GAPP scores of nonsurvivors was too small, however, to allow statistical comparisons. Details of radiological imaging results, surgical approaches, experience of surgeons, duration of surgery, PASS and GAPP-score, decades of surgery and years of last FU of survivors or death of nonsurvivors, as appropriate, are given in Suppl. Table 6.

3.4 Metastatic and nonmetastatic recurrences

Twelve patients (3.4% of the study cohort, 6 uPCC, 6 aPGL) had primary metastatic disease. Six had local lymph node and six distant metastases at first diagnosis. One patient (with positive lymph nodes at first surgery) is alive after a FU of 1.3 years, eleven died after a median FU of 4.8 (range 2.4-9.9) years.

There were 57 patients (18.8%) with recurrent disease, 24 with metastatic (7.9%) and 33 nonmetastatic recurrences (10.9%). Metastatic recurrences occurred in 12 of 231 (5.2%) survivors and in 12 of 72 (16.7%) nonsurvivors (p<0.0001). All metastases were at distant sites and progressed throughout the FU period. However, not all tumor progressions represented the cause of death (Suppl. Table 8). Nonmetastatic recurrences were observed in 28 survivors (12.1%) and in 5 nonsurvivors (6.9%) (p=0.008). Survivors were younger than nonsurvivors (p=0.002), the mean age of survivors with nonmetastatic recurrences lower than that of nonsurvivors with metastatic recurrences (29.6 vs. 46.4 years, p=0.03). There were more HNPGL in survivors than in nonsurvivors (p=0.03). The number of PCC and of PGL were not different between survivors and nonsurvivors. Positive family history was observed more often in patients with nonmetastatic than with metastatic recurrences (p=0.0005). Pathogenic germline variants and smaller tumor size were more common in survivors with nonmetastatic recurrence than in nonsurvivors with metastatic recurrence (p<0.0001 and p=0.0007, respectively). The last FU visit for 159 (68.9%) survivors was in 2023, for 40 (17.3%) in 2022. The proportion of patients with metastatic and nonmetastatic recurrences last seen in 2023 was not different. The small number of survivors last

seen before the 2020s (n=32, 13.8%) had their last FU visit with comparable frequency in the 1990s, 2000s and 2010s. Characteristics of patients with recurrent PPGL are given in Suppl. Tables 9 and 10.

3.5 Recurrence free survival (RFS) and post recurrence survival (PRS)

The probability of recurrence did not differ between patients with metastatic and nonmetastatic recurrence (median time to recurrence 31.3 years vs. undefined, HR 0.75, 95% CI 0.44-1.26, p=0.27) (Figure 5). The RFS and PRS were 9.0±8.0 and 2.0±5.9 years in 231 survivors and 6.8±6.6 and 2.7±6.6 years in 72 non-survivors, respectively (p=n.s. for all comparisons between survivors and nonsurvivors). Of the 57 patients with recurrent PPGL, the OAS, RFS and PRS were 6.9±6.7 years, 10.8±9.7 years and 17.8±10.5 years in the 40 survivors, 8.0±8.3 years, 8.7±9.6 years and 16.7±13.6 years in the 17 non-survivors (p=n.s. for all comparisons between survivors and nonsurvivors, Suppl. Tables 9 and 10).

When compared to patients with metastatic recurrence, however, those with nonmetastatic recurrence had better overall survival after the recurrence (median 37.0 vs. 11.1 years, HR 3.9, 95% CI 1.5-10.6, p=0.007), better PRS due to PPGL-related oncological disease (no post recurrence death vs. 9 of 24, median undefined vs. 6.2 years, HR 11.4, 95% CI 3.0-43.1, p=0.0003) and better PFS as defined by death due to PPGL- and CVD combined (median 37.0 vs. 10.4 years, HR 5.0, 95% CI 1.6-15.3, p=0.005) (Suppl. Figure 1).

3.6 Prognostic factors for recurrence-free survival (RFS)

Significant predictors of shorter RFS were younger age (HR 0.96, 95% CI 0.93-0.99), comorbid cardiovascular diasease (HR 4.0, 95% CI 1.7-9.2) and positive germline genetic results (HR 3.1, 95% CI 1.5-6.8), especially cluster 1A (HR 11.1, 95% CI 4.7-27.5), which corresponded predominantly to germline mutations in *SDHA-D* genes in our patients (Suppl. Table 13).

4. Discussion

4.3 Recurrence and prognostic implications

In our study, the recurrence rate was 13% (95% CI 18-18), 20% (95% CI 16-26) and 40% (95% CI 30-50) five, ten and twenty years after first surgery, respectively (Suppl. Figure 1) and is higher than reported previously. A recent systematic review and meta-analysis of recurrence rates after curative adrenalectomy included 13 studies and calculated a cumulative tumor recurrence of 1.9% (8 of 430 patients, 6 with metastatic recurrence). However, the duration of FU of the studies was short ranging from three to nine years and the median number of included patients per study was 22. Only one study evaluated more than 100, the other twelve a range of 14 to 41 patients (22). Another systematic review analyzed 53 cohorts, including 15 with HNPGL, with patients of one to 21 centers, most being followed by surgical teams (23). The cumulative median recurrence rate was 6% (range 1-31%), the 5-year cumulative incidence of recurrence 4.7% (95% CI 3.4-6.1) and the proportion of metastatic recurrences 55%. The authors tabulate details of 38 studies and report a median of 55 (range 22-239) patients per study. As with the first meta-analysis, data were scarce after the first ten years of FU, only four studies provided a clear and specific description of patient flow including FU duration, two studies had performed multivariate analyses, risk factors were not consistently assessed across the studies and only a few had available genetic data (23). A recent multicenter study of 398 patients from seven tertiary centers reported a recurrence rate of 47.5% of patients with cluster 1 (mainly metastatic), 14.9% with cluster 2 hereditary diseases and 14.7% of sporadic PPGL. 75 of 224 (33.5%) patients with sporadic disease had a previous history of PPGL. In a subanalysis of 149 patients without previous history of PPGL and a FU duration of at least five years, the recurrence rate was 23,1%, 11.1% and 6.3% of patients with cluster 1, cluster 2 and without genetic abnormalities, respectively. Three of 26 patients with cluster 1, none of 27 with cluster 2 and one of 96 with sporadic recurrent PPGL presented with metastatic recurrences (24). In our study, mean FU duration was more than eleven (ranging up to 50) years and >90% of patients had germline genetic data available. The total of 57 patients with recurrent PPGL (33 nonmetastatic, 24 metastatic) allowed a risk factor analysis by Cox regression with up to 6 dependent variables. Younger age, smaller tumor size, as well as cluster 1 and 2 pathogenic variants were predictive of nonmetastatic recurrence, larger tumor size, a first operation performed by nonspecialized surgeons predictive of metastatic recurrence. The heterogeneity of published studies limits the comparability of data. Larger tumor size, younger age, higher PASS score and positive genetic findings

have been reported in an Italian study of 177 patients with PCC from 9 centers. 74 (41.8%) had genetic tests performed, 40 were positive, predominantly *NF1* and *VHL* (25). Larger tumor size, extraadrenal tumor location and a non-adrenergic phenotype have been suggested to be predictive of recurrence of PPGL, derived from the data of 298 patients with sporadic PPGL enrolled at seven tertiary European and one quaternary US center, including 75 with a previous history of PPGL (24). Others have reported larger tumor size, the histopathological proliferation marker Antigen Kiel 67 (Ki67) ≥3% and *SDHB* pathogenic variants to be predictive of loco-regional recurrence, such as by a Chinese study of 96 patients followed for a median of six (range 4-9) years (26). Almost 20 years ago, larger tumor size and extra-adrenal tumor location vs. in the left adrenal were predictive of recurrence of 176 patients of a single-center study from France. Familial disease was evaluated on clinical grounds and a median FU of 9.1 (range 5.5-15.3) years presented (27). The recurrence rates reported vary not only due to genetic background (23), but also due to many other factors such as number of patients included, the database used, the FU duration, whether patients with primary metastases or with a previous history of PPGL were included, whether patients with benign or malignant disease (or with any combination) fell into the study design and whether patients only with PCC, with both PCC and PGL or with HNPGL were included. Insensitivity bias may have influenced the results of previous studies, as some driver genes for hereditary disease were not known in former years (see Suppl. Material on germline genetic analysis). Follow-up bias may overestimate recurrence rates, as patients with recurrence are more likely to attend FU examinations (28). Statistical issues such as overfitting may have been contributory as well. Asking more questions, than the data can answer occurs when too many independent variables in the predictive model are compared to the number of subjects or the dependent variables for the model are chosen after data have been gathered. The results may be large regression coefficients and small p-values, which will not be reproduced when tested on a new set of data (21,29). To overcome this problem, it is tempting, especially with a rare disease such as PPGL, to recruit patients from many centers or from a large database to increase sample size, the number of events and the number of dependent variables in the regression model. The statistical rule of thumb of ten events per variable may be met, yet comparability of data and generalizability to clinical practice become more limited.

In our study, probability of recurrence (the recurrence-free survival) was not different between 24 patients with metastatic vs. 33 with nonmetastatic recurrence. However, OAS, DSS1 and 2, as well as post-recurrence survival were worse in patients with metastatic compared to nonmetastatic recurrence, the median survival ranging from 19.9-21.2 vs. more than 38 years, respectively. Of note, no PPGL-

related deaths occurred in patients with nonmetastatic recurrence. Cluster 1B pathogenic variants were predictive of nonmetastatic, but not of metastatic recurrence. *SDHB* gene mutations have been associated with a greater propensity to metastatic recurrence and a worse overall survival in most (30-34), but not all (35,36) studies. In our unselected cohort, the number of patients with pathogenic *SDHB* variants was small (n=8), precluding the analysis of SDHB for recurrence risk. Six patients with *SDHB* mutations are alive after a median FU of 7.2 (range 1.0-31.2) years (one with metastatic, two with nonmetastatic recurrence and three without recurrence) and two died of PPGL 5.5 and 8.3 years after the first operation (Suppl. Table 7).

4.4 Discussion of Cox vs logistic regression analyses for evaluating prognostic factors for survival in patients with PPGL

Patients suffering from MACE were older than those without MACE (57.6±14.0 vs. 48.0±16.3 years, p=0.003). However, performing multivariable logistic regression to check for a confounding effect of age on MACE did not change the results of the Cox regression analysis in that MACE was the second most important independent risk factor for both the overall survival (OAS) and the disease-specific survival 2 (DSS 2).

Comparison of the results of both regression analyses for illustrative purpose are given in Supplementary Table 14. Please note, that the model presented is slightly different to that in Table 3 (genetic clusters included, sex not included) but comparable for both the logistic and the Cox regression analyses. Interestingly, the former method suggested tumor size and cluster II pathogenic variants as independent predictor of OAS which was not the case with the Cox regression. In addition, effect size of the independent variables in general was larger with the logistic regression than with the Cox regression, suggesting a greater specificity of the latter.

When recording actual time to event, the logistic regression coefficient will approximate that of the Cox model only when the follow-up period is short and the event rare (37). Our patients had a rather long follow-up, and the event was not rare (72 overall deaths of a cohort of 303, 23.8%). If the follow-up period is longer than 5 years, both methods are no longer comparable: the logistic regression coefficient increases while the Cox regression coefficient does not. In addition, the standard error estimate

in the Cox model is reduced compared with the logistic regression model as follow-up periods increase (38). Both, logistic and Cox regression are widely adopted methods for multivariate analysis. Cox regression is used when analyzing survival data over time, in dealing with time-to-event outcomes, while logistic regression is deemed appropriate for either-or clinical outcomes without the need to take into account the time until occurrence of the event of interest (39,40). In our opinion, the death of patients with PPGL should be regarded as a time-to-event rather than a either-or coutcome since time to death matters. Also, Cox regression due to the capability of managing censored data may be a better choice than logistic regression (41,42).

There have been several studies evaluating predictive factors for survival of patients with PPGL. Authors have not reached agreement regarding which regression model to use for multivarable analysis, however. Among the cited literature in our study, 14 studies opted for Cox regression (References 18, 21, 27, 31, 32, 56, 58-63, 74, 83 of the main manuscript) but only three for logistic regression (References 25, 29, 30 of the main manuscript). In our opinion, Cox regression rather than logistic regression is the more scientific approach to identify risk factors for death of patients with PPGL.

References

(1) Eisenhofer, G., Pamporaki, C., & Lenders, J. W. M. (2023). Biochemical Assessment of Pheochromocytoma and Paraganglioma. Endocrine Reviews, 44(5), 862–909. https://doi.org/10.1210/ENDREV/BNAD011

(2) Cascón, A., Calsina, B., Monteagudo, M., Mellid, S., Díaz-Talavera, A., Currás Freixes, M., & Robledo, M. (2023). Genetic bases of pheochromocytoma and paraganglioma. Journal of Molecular Endocrinology, 70(3). https://doi.org/10.1530/JME-22-0167

(3) Ponder, B. (1990). Neurofibromatosis gene cloned. Nature 1990 346:6286, 346(6286), 703–704. https://doi.org/10.1038/346703a0

(4) Mulligan, L. M., Kwok, J. B. J., Healey, C. S., Elsdon, M. J., Eng, C., Gardner, E., Love, D. R., Mole, S. E., Moore, J. K., Papi, L., Ponder, M. A., Telenius, H., Tunnacliffe, A., & Ponder, B. A. J. (1993). Germ-line mutations of the RET proto-oncogene in multiple endocrine neoplasia type 2A. Nature, 363(6428), 458–460. https://doi.org/10.1038/363458A0

(5) Latif, F., Tory, K., Gnarra, J., Yao, M., Duh, F. M., Orcutt, M. lou, Stackhouse, T., Kuzmin, I., Modi, W., Geil, L., Schmidt, L., Zhou, F., Li, H., Wei, M. H., Chen, F., Glenn, G., Choyke, P., Walther, M. M., Weng, Y., … Lerman, M. I. (1993). Identification of the von Hippel-Lindau disease tumor suppressor gene. Science (New York, N.Y.), 260(5112), 1317–1320. https://doi.org/10.1126/SCIENCE.8493574

(6) Baysal, B. E., Ferrell, R. E., Willett-Brozick, J. E., Lawrence, E. C., Myssiorek, D., Bosch, A., van der Mey, A., Taschner, P. E. M., Rubinstein, W. S., Myers, E. N., Richard, C. W., Cornelisse, C. J., Devilee, P., & Devlin, B. (2000). Mutations in SDHD, a mitochondrial complex II gene, in hereditary paraganglioma. Science (New York, N.Y.), 287(5454), 848–851. https://doi.org/10.1126/SCIENCE.287.5454.848

(7) Niemann, S., & Muller, U. (2000). Mutations in SDHC cause autosomal dominant paraganglioma, type 3. Nature Genetics, 26(3), 268–270. https://doi.org/10.1038/81551

(8) Astuti, D., Latif, F., Dallol, A., Dahia, P. L. M., Douglas, F., George, E., Sköldberg, F., Husebye, E. S., Eng, C., & Maher, E. R. (2001). Gene mutations in the succinate dehydrogenase subunit SDHB

cause susceptibility to familial pheochromocytoma and to familial paraganglioma. American Journal of Human Genetics, 69(1), 49–54. https://doi.org/10.1086/321282

(9) Fink', M., Weinhausel', A., Niederle~, B., & As', O. A. H. (1996). DISTINCTION BETWEEN SPORADIC AND HEREDITARY MEDULLARY THYROID CARCINOMA (MTC) BY MUTATION ANALYSIS OF THE RET PROTO-ONCOGENE. Int. J. Cancer Pred. Oncol, 312–316. https://doi.org/10.1002/(SICI)1097-0215(19960822)69:4

(10) Neumayer, C., Moritz, A., Asari, R., Weinhäusel, A., Hölzenbein, T., Kretschmer, G., Niederle, B., & Haas, O. A. (2007). Novel SDHD germ-line mutations in pheochromocytoma patients. European Journal of Clinical Investigation, 37(7), 544–551. https://doi.org/10.1111/J.1365-2362.2007.01822.X

(11) Flicker, K., Ulz, P., Höger, H., Zeitlhofer, P., Haas, O. A., Behmel, A., Buchinger, W., Scheuba, C., Niederle, B., Pfragner, R., & Speicher, M. R. (2012). High-resolution analysis of alterations in medullary thyroid carcinoma genomes. International Journal of Cancer, 131(2). https://doi.org/10.1002/IJC.26494

(12) Vierhapper, H., Bieglmayer, C., Heinze, G., & Baumgartner-Parzer, S. (2004). Frequency of RET proto-oncogene mutations in patients with normal and with moderately elevated pentagastrin-stimulated serum concentrations of calcitonin. Thyroid : Official Journal of the American Thyroid Association, 14(8), 580–583. https://doi.org/10.1089/1050725041692990

(13) GATK. (n.d.). Retrieved Jaunary 18, 2024, from https://gatk.broadinstitute.org/hc/en-us

(14) gnomAD. (n.d.). Retrieved January 18, 2024, from https://gnomad.broadinstitute.org/

(15) Home - OMIM. (n.d.). Retrieved January 18, 2024, from https://www.omim.org/

(16) ClinVar - ClinGen | Clinical Genome Resource. (n.d.). Retrieved January 18, 2024, from https://www.clinicalgenome.org/data-sharing/clinvar/

(17) LOVD - An Open Source DNA variation database system. (n.d.). Retrieved January 18, 2024, from https://www.lovd.nl/

(18) Richards, S., Aziz, N., Bale, S., Bick, D., Das, S., Gastier-Foster, J., Grody, W. W., Hegde, M., Lyon, E., Spector, E., Voelkerding, K., & Rehm, H. L. (2015). Standards and guidelines for the

interpretation of sequence variants: a joint consensus recommendation of the American College of Medical Genetics and Genomics and the Association for Molecular Pathology. Genetics in Medicine: Official Journal of the American College of Medical Genetics, 17(5), 405–424. https://doi.org/10.1038/GIM.2015.30

(19) Human Genome Assembly GRCh38 - Genome Reference Consortium. (n.d.). Retrieved January 18, 2024, from https://www.ncbi.nlm.nih.gov/grc/human/data?asm=GRCh38

(20) Ellard, S., Baple, E. L., Callaway, A., Berry, I., Forrester, N., Turnbull, C., Owens, M., Eccles, D. M., Abbs, S., Scott, R., Deans, Z. C., Lester, T., Campbell, J., Newman, W. G., Ramsden, S., & Mcmullan, D. J. (2020). ACGS Best Practice Guidelines for Variant Classification in Rare Disease 2020 Recommendations ratified by ACGS Quality Subcommittee on 4 th. https://doi.org/10.1101/531210

(21) 33. Motulsky H. Chapter 38. Logistic and Proportional Hazards Regression, pp. 395 ff. In: Intuitive Biostatistics. A Nonmathematical Guide to Statistical Thinking. 4th Ed. 2018. Oxford University Press. New York. Oxford.

(22) Holscher I, Van Den Berg TJ, Dreijerink KMA, et al. Recurrence Rate of Sporadic Pheochromocytomas After Curative Adrenalectomy: A Systematic Review and Meta-analysis. J Clin Endocrinol Metab 2021;106(2):588–597.

(23) Amar L, Lussey-Lepoutre C, Lenders JWM, et al. MANAGEMENT OF ENDOCRINE DISEASE: Recurrence or new tumors after complete resection of pheochromocytomas and paragangliomas: a systematic review and meta-analysis. Eur J Endocrinol 2016;175(4):R135–R145.

(24) Li M, Prodanov T, Meuter L, et al. Recurrent Disease in Patients With Sporadic Pheochromocytoma and Paraganglioma. J Clin Endocrinol Metab 2023;108(2):397–404.

(25) Parasiliti-Caprino M, Bioletto F, Lopez C, et al. Development and internal validation of a predictive model for the estimation of pheochromocytoma recurrence risk after radical surgery. Eur J Endocrinol 2022;186(3):399–406.

(26) Kim JH, Moon H, Noh J, Lee J, Kim SG. Epidemiology and Prognosis of Pheochromocytoma/Paraganglioma in Korea: A Nationwide Study Based on the National Health Insurance Service. Endocrinol Metab (Seoul) 2020;35(1):157–164.

(27) Amar L, Servais A, Gimenez-Roqueplo AP, et al. Year of diagnosis, features at presentation, and risk of recurrence in patients with pheochromocytoma or secreting paraganglioma. J Clin Endocrinol Metab 2005;90(4):2110–2116.

(28) Plouin PF, Chatellier G, Fofol I, Corvol P. Tumor recurrence and hypertension persistence after successful pheochromocytoma operation. Hypertension 1997;29(5):1133–1139.

(29) Babyak MA. What you see may not be what you get: a brief, nontechnical introduction to overfitting in regression-type models. Psychosom Med 2004;66(3):411–421.

(30) Araujo-Castro M, García Sanz I, Mínguez Ojeda C, et al. Local recurrence and metastatic disease in pheochromocytomas and sympathetic paragangliomas. Front Endocrinol (Lausanne) 2023;14. doi:10.3389/fendo.2023.1279828.

(31) Cui Y, Ma X, Gao Y, et al. Local-Regional Recurrence of Pheochromocytoma/Paraganglioma: Characteristics, Risk Factors and Outcomes. Front Endocrinol (Lausanne) 2021;12. doi:10.3389/FENDO.2021.762548.

(32) Taïeb D, Nölting S, Perrier ND, et al. Management of phaeochromocytoma and paraganglioma in patients with germline SDHB pathogenic variants: an international expert Consensus statement. Nat Rev Endocrinol 2023. doi:10.1038/S41574-023-00926-0.

(33) Donato S, Simões H, Pinto AT, et al. SDHx-related pheochromocytoma/paraganglioma - genetic, clinical, and treatment outcomes in a series of 30 patients from a single center. Endocrine 2019;65(2):408–415.

(34) Neumann HPH, Pawlu C, Pęczkowska M, et al. Distinct clinical features of paraganglioma syndromes associated with SDHB and SDHD gene mutations. JAMA 2004;292(8):943–951.

(35) Hescot S, Curras-Freixes M, Deutschbein T, et al. Prognosis of Malignant Pheochromocytoma and Paraganglioma (MAPP-Prono Study): A European Network for the Study of Adrenal Tumors Retrospective Study. J Clin Endocrinol Metab 2019;104(6):2367–2374.

(36) Pamporaki C, Prodanov T, Meuter L, et al. Determinants of disease-specific survival in patients with and without metastatic pheochromocytoma and paraganglioma. Eur J Cancer 2022;169:32–41.

(37) Green MS, Symons MJ, A comparison of the logistic risk function and the proportional hazards model in prospective epidemiological studies. J Chron Dis. 1983; 36: 715-724).

(38) Hosmer DW, Lemeshow S, May R. Applied Survival Analsis: Regression Modeling of Time to Event Data. Second Edition 2008, Wiley, New York ,USA

(39) Guller U, DeLong ER. Interpreting statistics in medical literature: a vade mecum for surgeons. J Am Coll Surg. 2004; 198: 441-458

(40) van der Net JB, Janssens AC, Eijkemans MJ, et al. Cox proportional hazards models have more statistical power than logistic regression models in cross-sectional association studies. Eur J Hum Genet. 2008; 16: 1111-1116

(41) Kang H, Weidong T. Which to select when evaluating risk factors for permanent stoma, Cox regression model or logistic regression model? Am Transl Med. 2021; 9: 1634-1636

(42) George B, Seals S, Aban I. Survival analysis and regression models. J Nucl Cardiol. 2014; 21:686-694

(43) Vittinghoff E, McCulloch CE. Relaxing the rule of ten per variable in logistic and Cox regression. Am J Epidemiol. 2007; 165: 710-718).

Supplementary Figures

Supplementary Figure 1. Probability of recurrence of all patients at risk for recurrence, e.g. excluding those with metastatic disease at time zero.

Supplementary Figure 2. Overall survival (top), disease-specific survival 1, DSS1 (middle) and DSS2 (bottom) after recurrence (post-recurrence survival) of 57 patients with metastases-free PPGL after first diagnosis.

Supplementary Tables

Supplementary Table 1: Detailed characteristics of incidentally discovered patients.

Supplementary Table 2: Details of patients with major adverse cardiovascular events (MACE).

Supplementary Table 3: Detailed comorbidities.

Supplementary Table 4: Baseline characteristics, continued from Table 1 of the main text.

Supplementary Table 5: Details of germline analyses in the 4 genetic labs.

Supplementary Table 6: Predictive factors for survival of the total cohort (univariate analysis).

Supplementary Table 7: Details of patients with pathogenic germline variants.

Supplementary Table 8: Details of causes of death of 72 nonsurvivors.

Supplementary Table 9: Details of patients with primary metastatic and recurrent PPGL.

Supplementary Table 10: Details of patients with metastatic and nonmetastatic recurrence.

Supplementary Table 11: Number of deaths due to PPGL, CVD, and malignant + other diseases during the study period.

Supplementary Table 12: Predictive factors for overall and disease-specific survival of n=291 patients without metastatic disease at the begin of the study.

Supplementary Table 13. Predictive factors for recurrence-free survival of patients with metastatic (n=24) and nonmetastatic (n=33) recurrences of PPGL.

Supplementary Table 14. Comparison of multivariable analyses of predictive factors by Cox regression (presenting hazard ratios, HR) and logistic regression (presenting odds ratios, OR).

Supplementary Figure 1. Probability of recurrence of all patients at risk for recurrence, e.g. those with nonmetastatic disease at time zero (upper graph), with metastatic (dark shading) and nonmetastatic recurrence (light shading) depicted by the lower graph, respectively. Number (#) at risk beneath the graphs denote the number of patients at risk to be censored due to death or loss to FU at the start of the study and after each of the given 5-year intervals.

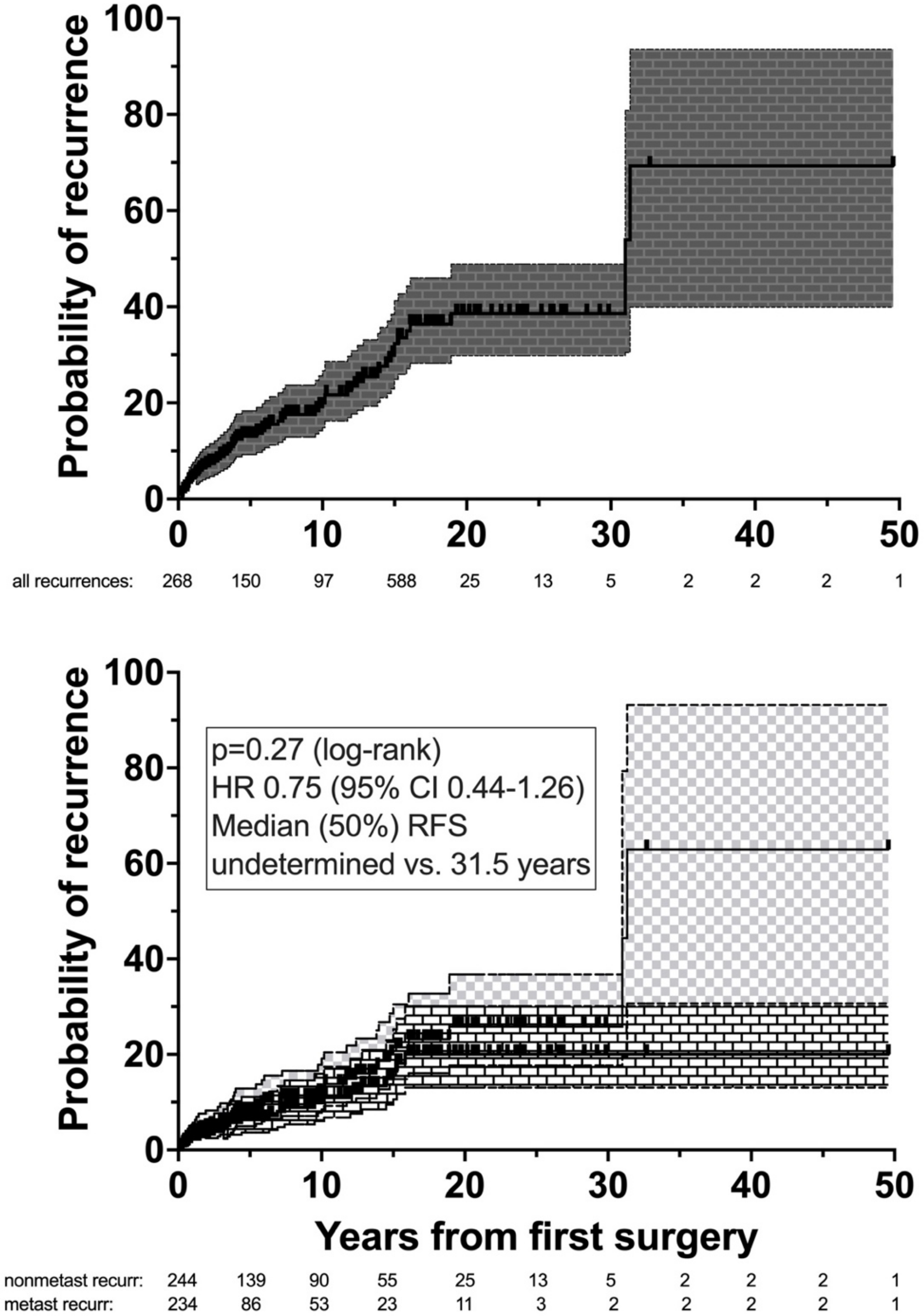

Supplementary Figure 2. Overall survival (top), DSS1 (middle) and DSS2 (bottom) after recurrence of 57 patients with metastases-free PPGL after first diagnosis (dark shading = patients with metastatic recurrences, light shading = those with nonmetastatic recurrences. Number (#) at risk beneath the graphs denote the number of patients at risk to be censored due to death or loss to FU at the start of the study and after each of the given 5-year intervals.

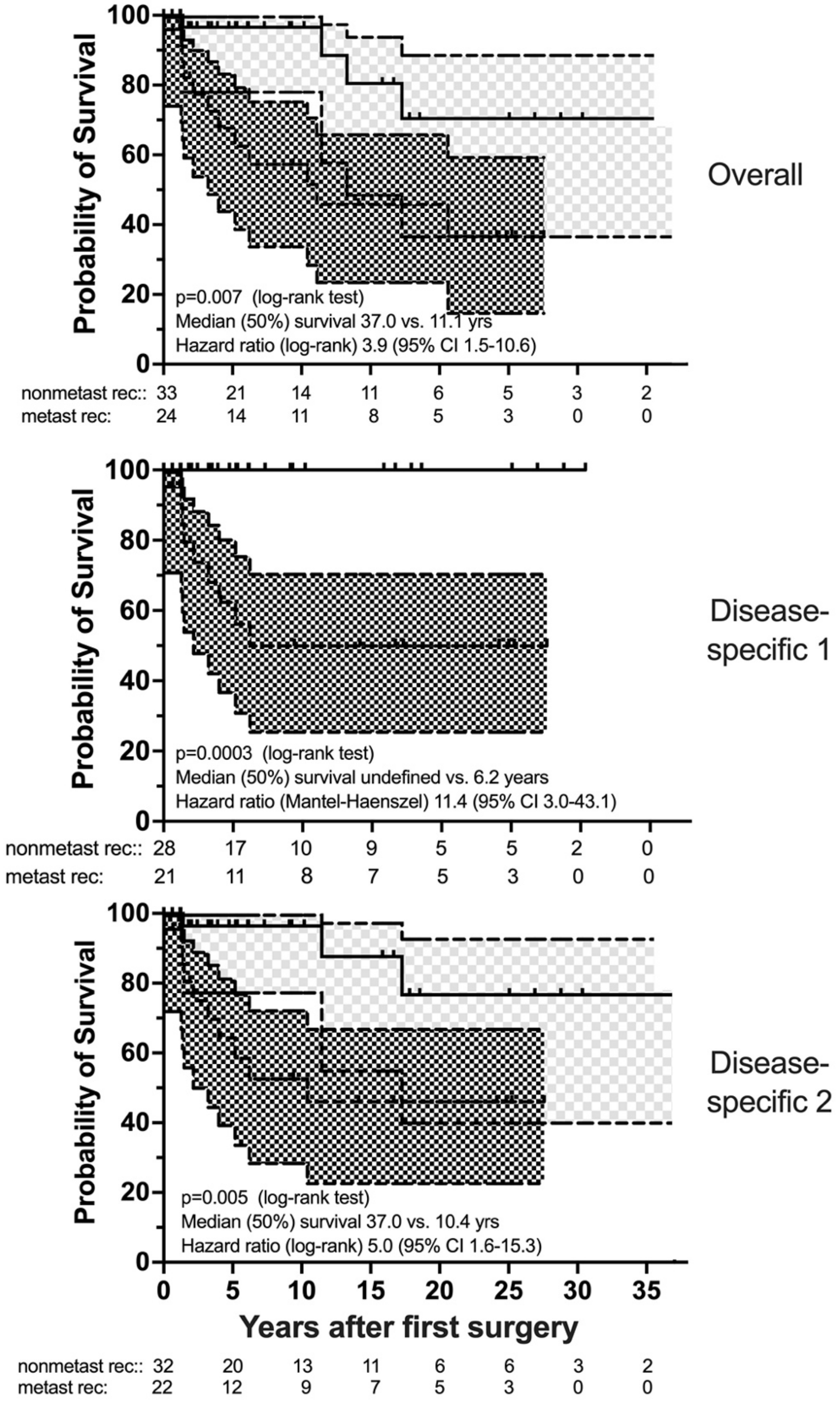

Supplementary Table 1. Details of 103 patients with incidentally diagnosed PPGL including age, sex(f=female, m=male), decades of surgery (DOS), histopathological results (Histo), cause of death, overall survival (OAS), primary metastatic disease and recurrence. Age and OAS are given in years at and after first surgery, respectively. Abbreviations not used in the text: Mal+ = malignant disease and others (not CVD, not COPD), US=ultrasound, AS = aortic stenosis, AAA= abdominal aortic aneurysm, CKD=chronic kidney disease, compl = complaints, abnormal = abnormalities.

Pat.ID	Age	Sex	DOS	Incident	Histo	Death	OAS	Prim meta	Recurrence
252	67,21	f	2000s	Acute heart failure	uPCC	CVD	19,92	no	metast (liver, lungs, Lnn)
80	59,67	f	2010s	Acute pancreatitis	uPCC		5,60		
140	68,30	f	2020s	After HNPGL	HNPGL		0,75		
246	55,00	m	2020s	After MTC	bPCC		0,93		
40	75,11	f	1990s	After surgery for AAA	uPCC	CVD	4,22		
16	58,72	f	2020s	Back pain	uPCC		1,91		
24	72,34	f	2000s	Back pain	uPCC		17,86		
104	43,17	m	2010s	Back pain	uPCC	CVD	8,74		
214	68,64	m	2000s	Back pain	uPCC	Mal+	13,67		
293	34,35	f	2000s	Back pain	uPCC		18,72		
162	44,30	f	2010s	Before bariatric surgery	uPCC		4,48		
64	46,01	m	2010s	Before renal transplant	uPCC		3,96		
69	62,73	m	2010s	Before surgery for AAA	uPCC	CVD	3,56		
172	84,37	f	2010s	Before surgery for AS	uPCC	CVD	3,04		
253	54,55	f	2000s	Before surgery for ovarian cysts	uPCC		19,34		
29	65,07	m	1990s	Car accident	tPGL	PPGL	8,59	no	metast (local Lnn, multiple
160	47,99	m	2010s	Car accident	uPCC		4,08		
84	20,69	m	2010s	Chronic pancreatitis	uPCC		11,31		
266	38,51	m	2000s	Chronic pancreatitis	cPCC		8,25		
59	66,32	f	2010s	CKD, Anorexia	uPCC		8,29		
78	43,94	m	2010s	Diabetes	uPCC		9,84		
206	63,66	m	2000s	Diabetes	uPCC		17,78		
251	45,99	m	2000s	Diabetes	aPGL		17,50		
289	43,78	f	2000s	Diabetes	uPCC		14,65		
6	49,16	m	2020s	FU angioma of cervical spine	uPCC		0,63		
242	55,36	f	2000s	FU breast cancer	cPCC		16,40		
261	68,19	f	2020s	FU duodenal ulcer	cPCC		0,26		
235	77,16	f	2000s	FU longstanding adr tumor	uPCC	Mal+	12,66		
287	69,20	m	2010s	FU longstanding adr tumor	uPCC		5,38		
156	74,87	f	2010s	FU melanoma	uPCC		6,06		
296	63,66	m	2010s	FU of mononucleosis	uPCC		6,10		
194	39,79	m	2020s	FU of NF1	uPCC		1,19		
63	62,10	f	2000s	FU plasmocytoma	uPCC		2,24		
241	44,01	m	2000s	FU stomach cancer	uPCC	Mal+	1,73		
138	54,79	m	2010s	Gall stones	uPCC		4,10		
150	61,41	m	2010s	Gall stones	aPGL		9,85		
202	74,04	m	2000s	Gall stones	uPCC		3,01		
271	72,04	m	2010s	Gall stones	uPCC		0,27		
217	72,84	m	2000s	Hernia inguinal	cPCC		17,66		

Pat.ID	Age	Sex	DOS	Incident	Histo	Death	OAS	Prim meta	Recurrence
67	60,19	m	2020s	Kidney stones	uPCC		2,03		
229	52,00	m	2010s	Kidney stones	uPCC		7,30		
259	46,45	f	1990s	Lipoma inguinal	uPCC		24,03		
41	72,74	m	2000s	Pneumonia	uPCC	CVD	7,02		
215	51,58	f	1990s	Pneumonia	aPGL		8,17		
294	44,42	m	2010s	Pneumonia	aPGL		4,40	no	metast (local Lnn, multiple
230	72,05	f	1990s	Primary hyperpara	bPCC	CVD	7,16		
11	55,69	f	2000s	Routine abd US	uPCC		1,79		
45	41,22	f	2010s	Routine abd US	uPCC		11,24		
66	37,23	f	2000s	Routine abd US	uPCC		16,73		
105	38,53	f	2000s	Routine abd US	uPCC		15,45		
112	76,18	f	2010s	Routine abd US	uPCC		4,45		
113	61,31	f	2000s	Routine abd US	aPGL	CVD	9,88	yes (Lnn)	progress, but not cause of death
126	76,71	m	2020s	Routine abd US	uPCC		0,85		
147	70,82	f	2020s	Routine abd US	aPGL		1,00	no	metast (liver, lungs)
154	54,72	f	1990s	Routine abd US	uPCC		25,19		
176	52,63	m	1990s	Routine abd US	uPCC	Mal+	15,78		
282	46,01	f	2010s	Routine abd US	uPCC		6,12		
290	64,50	f	2020s	Routine abd US	uPCC		0,41		
163	42,13	f	2010s	Unclear anemia	aPGL		0,70		
164	25,67	f	2010s	Unclear anemia	HNPGL		5,72	no	nonmetast (tPGL)
72	49,47	f	2010s	Unclear ankle pain	cPCC		10,25		
260	46,68	f	2010s	Unclear diarrhea	uPCC		12,50		
234	73,21	f	2010s	Unclear dizziness	uPCC		7,65		
74	63,95	f	2010s	Unclear epigastric compl	aPGL		1,28		
110	51,29	m	2010s	Unclear epigastric compl	uPCC		13,35		
132	24,67	f	1990s	Unclear epigastric compl	uPCC		29,23		
197	43,75	f	1990s	Unclear epigastric compl	uPCC		26,01		
201	49,85	f	2000s	Unclear epigastric compl	aPGL	Mal+	15,26		
205	77,14	f	2010s	Unclear epigastric compl	uPCC		5,60		
240	66,77	m	2000s	Unclear epigastric compl	uPCC		17,18		
298	65,76	f	2020s	Unclear epigastric compl	uPCC		1,44		
157	75,03	m	2020s	Unclear hematuria	bPCC		2,46		
227	60,22	m	2010s	Unclear hematuria	uPCC		10,28		
244	54,50	f	2020s	Unclear liver function abnormal	uPCC		3,50		
291	56,66	f	2010s	Unclear liver function abnormal	uPCC		2,68		
25	72,86	m	2010s	Unclear lower abd compl	uPCC		9,04		
54	66,21	f	2010s	Unclear lower abd compl	uPCC	PPGL	3,48	no	metast (distant Lnn, mult bones)
127	30,28	m	1990s	Unclear lower abd compl	uPCC	PPGL	0,55	no	metast (liver, lungs, mult bones)
146	66,23	f	2000s	Unclear lower abd compl	uPCC	Mal+	12,29		
299	56,78	f	2010s	Unclear lower abd compl	uPCC		4,19		
101	39,27	m	2000s	Unclear nausea	bPCC		22,52		
239	63,69	f	2020s	Unclear polyneuropathy	uPCC		1,06		
161	27,30	m	1990s	Unclear thoracal compl	uPCC		22,38		
269	68,18	f	2000s	Unclear thoracal compl	uPCC	Mal+	13,67		

Pat.ID	Age	Sex	DOS	Incident	Histo	Death	OAS	Prim meta	Recurrence
18	59,83	f	2010s	Unclear urinary incontinence	uPCC		9,81		
62	32,35	f	2000s	Unclear vaginal bleeding	mPPGL		17,50		
233	52,10	f	2020s	Unclear weight loss	uPCC		2,08	no	metast (liver, lungs, Lnn)
213	63,15	f	2010s	US supraaortic art	mPPGL		4,59		
57	54,62	f	2010s	Weight loss	uPCC		9,61		

Supplementary Table 2. Clinical characteristics and survival (overall and disease specific) of 44 patients (14.5% of the total cohort) with major adverse cardiovascular events (MACE) prior to first surgery (n=42) or during biochemical progression (n=2) of PPGL, either leading (n=18) or not leading (n=26) to the diagnosis of PPGL. There were 5 patients suffering from a second MACE (MACE 2) 5, 12, 18, 26 and 35 months after the first (MACE 1 and 2 given as months prior to first surgery). The time between MACE and first surgery of patients with MACE leading to the diagnosis of PPGL was not different to those with incidental diagnosis despite prior MACE (10.8±17.3 vs. 14.4±17.1 months, p=0.84). Abbreviations not explained and used in the text: coron=coronary, syndr=syndrome, CeV=cerebrovascular, AMI=acute myocardial infarction, NSTEMI=non ST-elevation myocardial infarction, STEMI=ST-elevation myocardial infarction, TIA=transient ischemic attack, AoAneu=aortic aneurysm, PRIND=prolonged reversible ischemic deficit, abd=abdominal, compl=complaints, chron diarrh=chronic diarrhea, wt=weight, w/ = with, PAD=peripheral arterial vascular disease, DVT=deep vein thrombosis, PE=pulmonary embolism, iCMP=ischemic cardiomyopathy, HE=hysterectomy, CKD=chronic kidney disease, AAA=abdominal aortic aneurysm,bar surg=bariatric surgery, RCC=renal cell carcinoma, NSCLC=non small-cell lung cancer, Ca=carcinoma, adr tu=adrenal tumor.

Pat-ID	Age	Sex	Diagnosis due to	MACE	1st OP	Death	Cause	MACE	MACE	Comment	Incident
26	70	female	Incidental desp MACE	Acute coron syndr, AMI, not further specified	2004	2006	CVD	6		*(a)*	abd imaging for abdom compl
102	62	female	Incidental desp MACE	Acute coron syndr, AMI, not further specified	2021			40			abd imaging for chron diarrh+wt loss
168	47	female	Incidental desp MACE	Acute coron syndr, AMI, not further specified	2010			10		*(b)*	abd imaging for abdom compl
177	53	male	Incidental desp MACE	Acute coron syndr, AMI, not further specified	2011			12			abdom imaging w/PAD
117	55	female	Incidental desp MACE	Acute coron syndr, AMI, not further specified, and Takotsubo	2014			40			abd imaging w/DVT+PE
169	68	female	Incidental desp MACE	Acute coron syndr, AMI, not further specified, and Takotsubo	2013			4		*(c)*	abd imaging for abdom compl
280	42	female	Incidental desp MACE	Acute coron syndr, AMI, not further specified, and Takotsubo	2010			60	44		abdom imaging post AMI
159	72	male	Incidental desp MACE	Acute coron syndr, not further specified	2007	2018	CVD	0.5			abdom imaging w/iCMP+PAD
181	79	male	Incidental desp MACE	Acute coron syndr, not further specified (2x)	2023			29	3	*(d)*	abdom imaging for renal stones
131	72	female	Incidental desp MACE	Acute coron syndr, NSTEMI	2021			2			abdom imaging for dyspnea+angina
114	77	female	Incidental desp MACE	Acute coron syndr, STEMI	2002	2014	CVD	2			uncontrolled hypertension
270	74	male	Incidental desp MACE	Acute CeV event, PRIND	2001	2004	Mal	0,2			abdom imaging for falls
53	55	female	Incidental desp MACE	Acute CeV event, stroke, not further specified	2008			30			abd imaging post HE
60	44	male	Incidental desp MACE	Acute CeV event, stroke, not further specified	2023			6			abd imaging w/CKD, anorexia
243	61	male	Incidental desp MACE	Acute CeV event, stroke, not further specified	2002			6			abd imaging for abdom compl
119	45	female	Incidental desp MACE	Acute CeV event, stroke, not further specified - AMI, STEMI	2016			44	9		abdom imaging for abdom compl
303	76	male	Incidental desp MACE	Acute CeV event, stroke, not further specified (2x)	2000	2007	PPGL	24	12		abdom imaging w/AAA
190	49	female	Incidental desp MACE	Acute CeV event, TIA	2015	2017	Mal	5			abdom imaging post bar surg
130	59	male	Incidental desp MACE	Cardiac arrest, in-hospital	2008	2021	CVD	1			uncontrolled hypertension

Pat-ID	Age	Sex	Diagnosis due to	MACE	1st OP	Death	Cause	MACE	MACE	Comment	Incident
166	47	female	Incidental desp MACE	Critical lower limb ischemia, "leg for life" surgery	2004	2020	Mal	3			abdom imaging w/DVT
92	72	female	Incidental desp MACE	Dissecting AoAneu	1992	1992	Mal	1			abd imaging post RCC+NSCLC
75	23	male	Incidental desp MACE	Life-threatening bleeding	1983					*(e)*	PGL not thought of
90	68	male	Incidental desp MACE	Pulmonary edema	1994	2006	CVD	21			abd imaging post bladder Ca
188	54	female	Incidental desp MACE	Takotsubo	2021			3			abdom imaging for abdom compl
1	57	male	RR crisis despite	Acute coron syndr, STEMI	2010			0,5			uncontrolled hypertension
13	64	male	RR crisis despite	Pulmonary edema	2012			4		*(f)*	abdom imaging w/adr tu
136	73	female	MACE	Acute coron syndr, AMI, not further specified	1994	2015	CVD	4			
170	57	female	MACE	Acute coron syndr, AMI, not further specified	2004	2013	CVD	10		*(g)*	
171	64	female	MACE	Acute coron syndr, AMI, not further specified	2020			11			
17	52	female	MACE	Acute coron syndr, NSTEMI	1988					*(h)*	
198	37	male	MACE	Acute coron syndr, STEMI	1991					*(i)*	
125	56	male	MACE	Acute CeV event, PRIND	2019	2022	PPGL	7	2		
77	52	male	MACE	Acute CeV event, stroke, not further specified	1980	1996	CVD	6			
100	67	male	MACE	Acute CeV event, stroke, not further specified	2005	2019	Mal	6			
143	71	male	MACE	Acute CeV event, stroke, not further specified	2021			6			
151	44	male	MACE	Acute CeV event, stroke, not further specified	1995	2004	Mal	3			
216	80	male	MACE	Acute CeV event, stroke, not further specified	2012	2016	Mal	24			
103	68	female	MACE	Acute CeV event, TIA	2004	2019	CVD	3			
204	73	female	MACE	Acute CeV event, TIA	2000	2004	CVD	1			
210	47	male	MACE	Acute CeV event, TIA	1994	1997	PPGL	1			
219	44	male	MACE	Acute CeV event, TIA	2000	2003	CVD	5			
180	36	female	MACE	Cardiac arrest w/ acute cv event, stroke, not further specified	1998			12			
70	35	female	MACE	Eklampsia	2005			60			
267	37	female	MACE	Takotsubo	2020			2			

(a) adr tu known for 3 yrs, (b) adr tu known for 10 yrs before MACE
(c) adr tu known for 7 yrs before MACE
(d) adr tu known for 1 yrs before 2nd MACE
(e) MACE at surgery/HNPGL
(f) adr tu known for 7 yrs before MACE
(g) adr tu known for 5 yrs before MACE
(h) MACE 24 yrs after 1st OP w/biochem progress (metast rec)
(i) MACE 31.5 yrs after 1st OP w/biochem progress (nonmetast rec)

Supplementary Table 3. Details of comorbidities (groups 0=no comorbidity, 1= at least two classical CV risk factors (RF), 2= established CVD, 3=malignant disease without CV RF, 4= malignant disease with CV RF, 5=malignant disease with established CVD, 6=others) before first diagnosis of 303 patients with PPGL. Numbers do not sum up to 100%, as most patients suffered from more than one of the listed diseases. 2 co-morbid details denote those of group two, 3 co-morbid details of group three and 6 co-morbid details of other diseases, respectively. Details for group 5 are tabulated for all 19 patients, a summary given for the other groups. Abbreviations not used in the text: CAD=coronary artery disease, CeVD=cerebrovascular disease, PAD=peripheral artery disease, CMP=cardiomyopathy, AF=atrial fibrillation, PE=pulmonary embolism, COPD=chronic obstructive pulmonary disease, SAH=subarachnoid hemorrhage, Ao/Mi valve dis=aortic or mitral valve disease, CKD=chronic kidney disease (insufficiency), AoAneu=aortic aneurysm, NSCLC=non small-cell lung cancer, SCLC=small-cell lung cancer, RCC=renal cell carcinoma, Ca = carcinoma, MDS=myelodysplastic syndrome, CNS=central nervous system, NET=neuroendocrine tumor, LTX=lung transplantation, diss =dissection, PTC=papillary thyroid carcinoma, MGUS=monclonal gammopathy of unknown significance.

Comorb	Surv, n	Nonsurv, n	PPGL	CVD	Mal+	2 Co-Morbid details	3 Co-Morbid details	6 Co-Morbid details
0	37	7	5	1	1			
1*	98	9	4	1	4	Endocarditis		Migraine, Epilepsy, MS<br>Depresssion (3), BDZ abuse<br>Psychosis, Alcoholism (2)<br>Polytoxicomania<br>Perityphilitic abscess<br>Bariatr surg, bronchial asthma
2	50	31	4	21	6	CAD (43), CeVD (16)<br>both (8), CMP (6), PAD (11)<br>AF (7), PE (9), COPD (8)<br>Takotsubo (3), SAH (2)<br>Ao/Mi valve dis (10)<br>CKD (4), AoAneu (2)		Bariatr surg w/complic<br>Depression, FV Leiden+AT II def
3	15	7	0	0	7		MTC (11), NSCLC (3)<br>AML, Hodgkin<br>RCC, Melanoma,<br>Ca/Colon, Ca/Pancreas<br>Ca/Stomach, uterine sar-	
4	14	5	1	0	4		MTC (5), NSCLC (3),<br>Lymphoma (2), MDS<br>Melanoma, Ca/Breast (2)<br>CNS-hemangioblastoma	Depression (2), Sarkoidosis<br>MS, bronchial asthma

Ca/Uterus, NET/Pancreas

Comorb	Surv, n	Nonsurv, n	PPGL	CVD	Mal+	2 Co-Morbid details	3 Co-Morbid details	6 Co-Morbid details
5	8	11	1	5	5	CeVD, CMP	Ca/Bladder	Depression
5						CMP	Ca/Breast	Depression
5						CeVD	Ca/Colon	Osteoporose
5						CMP, CeVD, AF	Ca/Colon	
5						CeVD	Ca/Colon	
5						CeVD	Ca/Pancreas	
5						CAD, CeVD, PAD	Ca/Pancreas, MTC	
5						CeVD	Ca/Rectum	
5						CAD, CeVD, CMP, AF,	Ca/Rectum	
5						LTX	Ca/Uterus	
5						CAD, PAD	Ca/Uterus, MTC	
5						CAD, CMP	MTC	
5						CAD	MTC	
5						CAD	MTC	
5						CAD, CMP, AF	MTC	
5						PAD	Mult Myeloma	
5						AoAneu diss	NSCLC, RCC	
5						CAD, AF	PTC, T3	
5						PAD	RCC	
6	9	2	0	1	1			Bronchial asthma, Crohn´s dis
								Depression (2), migraine (2)
								Hydrocephalus, Parkinsson
								Portal vein thrombosis, MGUS
								Graves dis+renal oncozytoma

Supplementary Table 4. Characteristics of the study cohort (n=303 PPGL) followed for 50 years according to survivors (n=231) and nonsurvivors (n=72), continued from Table 1. *many patients were examined by more than one biochemical method simultaneously both preoperatively and during FU (the latter not displayed here). Thus, numbers sum up to more than 100%. Abbreviations are those used in the text.

	All	Survivors (n=231)	Non-survivors (n=72)	p-value
Family history				<0.0001
Positive, n (%)	48 (15.8)	42 (18.2)	6 (8.3)	
of which genetic results positive, n (%)	47 (97.9)	41 (97.6)	6 (100.0)	<0.0001 (pos vs unknown)
Cluster 1A, n	14	13	1	<0.0001 (neg vs unknown)
Cluster 1B, n	5	5	0	0.23 (pos vs neg)
Cluster 2, n	28	23	5	
of which genetic results negative, n (%)	1 (2.1)	1 (2.4)	0	
Negative, n (%)	228	179 (77.5)	49 (68.1)	
of which genetic results positive, n (%)	37 (16.2)	29 (16.2)	8 (16.3)	
Cluster 1A, n	10	9	1	
Cluster 1B, n	7	7	0	
Cluster 2, n	19	13	7	
of which genetic results negative, n (%)	174	144 (80.4)	30 (61.2)	
of which genetic results unknown, n (%)	17 (7.5)	6 (3.4)	11 (22.4)	
Unknown, n (%)	27 (8.9)	10 (4.3)	17 (23.6)	
of which genetic results positive, n (%)	11 (40.7)	6 (60.0)	5 (29.4)	
Cluster 1A, n	5	3	2	
Cluster 1B, n	2	2	0	
Cluster 2, n	4	1	3	
of which genetic results negative, n (%)	9 (33.3)	3 (30.0)	6 (35.3)	
of which genetic results unknown, n (%)	7 (25.9)	1 (10.0)	6 (35.3)	
Biochemical results				
Biochemical methods *				0.08
P-MNs, n (% of tested)	157	139 (67.5)	18 (38.3)	
U-MNs, n (% of tested)	132	111 (53.9)	21 (44.7)	
P-MNs+U-MNs simultaneously, n (% tested)	112	100 (48.5)	12 (25.5)	
U-CATs, n (% of tested)	218	174 (84.5)	44 (93.6)	

	All	Survivors (n=231)	Non-survivors	p-value
P-CATs, n (% of tested)	7 (2.8)	3 (1.5)	4 (8.5)	
Biochemically tested, n (%)	259	210 (90.9)	49 (68.1)	<0.0001
negative n (% tested)	18 (7.1)	17 (8.3)	1 (2.1)	
P-MNs contributing, n (% negative)	6	6	0	
U-MNs contributing, n (% negative)	1	1	0	
U-CATs contributing, n (% negative)	11	10	1	
P-CATs contributing, n (% negative)	0	0	0	
positive, n (% tested)	241	193 (83.6)	48 (98.0)	p<0.001 (P-MNs vs.
P-MNs contributing, n (% positive)	152	135 (70.0)	17 (35.4)	U-MNs + U-CATs)
U-MNs contributing, n (% positive)	21 (8.7)	12 (6.2)	9 (18.8)	
U-CATs contributing, n (% positive)	62 (25.7)	43 (22.3)	19 (39.6)	
P-CATs contributing, n (% positive)	6 (2.5)	5 (2.6)	1 (2.1)	
Biochemical parameters positive *				
P-NMN positive, n (% of P-MNs)	138	122 (87.8)	16 (88.9)	
P-MN positive, n (% of P-MNs)	114	103 (74.6)	11 (61.1)	
both, P-NMN + P-MN positive, n (% of P-MNs)	112	91 (65.5)	11 (61.1)	
U-NMN positive, n (% of U-MNs)	118	98 (88.3)	20 (95.2)	
U-MN positive, n (% of U-MNs)	114	85 (76.6)	19 (90.5)	
both, U-NMN + U-MN positive, n (% of U-MNs)	99 (75.0)	81 (73.0)	18 (85.7)	
U-NA positive, n (% of U-CATs)	136	106 (57.5)	30 (68.2)	
U-A positive, n (% of U-CATs)	122	95 (54.6)	27 (61.4)	
both, U-NA + U-A positive, n (% of U-CATs)	86 (39.4)	66 (37.9)	20 (45.5)	
P-NA positive, n (% of P-CATs)	7 (100.0)	3 (100.0)	4 (100.0)	
Biochemistry unknown, n (%)	50 (16.5)	25 (10.8)	25 (34.7)	
Radiological method				0.38
CT, n (%)	127	93 (40.3)	34 (47.2)	
MRI, n (%)	73 (24.1)	60 (26.0)	13 (18.1)	
CT+MRI, n (%)	77 (25.4)	60 (26.0)	17 (23.6)	
Ultrasound, n (%)	4 (1.3)	3 (1.3)	1 (1.4)	
Unknown, n (%)	22 (7.3)	15 (6.5)	7 (9.7)	
Functional imaging method				
F-DOPA PET-CT, n (%)	113	101 (43.7)	12 (16.7)	<0.0001
FDG PET-CT, n (%)	17 (5.6)	9 (3.9)	8 (11.1)	<0.0001 (FDOPA vs MIBG)

	All	Survivors (n=231)	Non-survivors (n=72)	p-value
MIBG, n (%)	70 (23.1)	44 (19.0)	26 (36.1)	0.0008 (F-DOPA vs FDG)
Unknown, n (%)	103	77 (33.3)	26 (36.1)	0.58 (FDG vs MIBG)
Surgical approach				<0.0001
Open, n (%)	114	70 (30.3)	44 (61.1)	
Laparoscopic transperitoneal, n (%)	142	119 (51.5)	23 (31.9)	<0.0001 (open vs. transperit)
Laparoscopic retroperitoneal, n (%)	42	41 (17.8)	1 (1.4)	<0.0001 (open vs. retroperit)
Transvaginal	1 (0.3)	1 (0.4)	0	0.02 (transperit vs. retroperit)
No surgery, including 1 Bx**, n (%)	4 (1.3)	0	4** (5.6)	
Duration of surgery, min (mean±SD)	149±92	182±87	209±88	<0.0001
Surgical experience				0.04
Specialized, n (%)	234	188 (81.3)	46 (63.9)	
Nonspecialized, n (%)	58 (19.1)	38 (16.5)	20 (27.8)	0.02 (special vs non-special)
Unknown, n (%)	7 (2.3)	5 (2.2)	2 (2.8)	
No surgery, n (%)	4 (1.3)	0	4 (5.6)	
Decades of first surgery				<0.0001
1980s (incl. 1 in 1968 and 2 in 1969*)	11 (3.6)	4*(1.7)	7* (9.7)	
1990s	54 (17.8)	33 (14.3)	21 (29.2)	0.0003 (1990s vs 2010s)
2000s	93 (30.7)	65 (28.1)	28 (38.9)	0.28 (1990s vs 2000s)
2010s	98 (32.3)	86 (37.2)	12 (16.7)	0.003 (2000s vs 2010s)
2020s	43 (14.2)	43 (18.6)	0	
No surgery (1990s: 2, 2010s: 2)	4 (1.3)	0	4 (5.6)	
PASS score, median (range), of n=214	2 (0-16)	1 (0-16)	3 (0-12)	
PASS <4, n (% of data available)	165/214	137/171 (80.1)	28/43 (62.8)	0.01
PASS ≥4, n (% of data available)	50/214	34/171 (19.9)	16/43 (37.2)	
GAPP score, median (range), of n=73	2 (0-8)	1 (0-8)	6 (1-6)	
GAPP <3, n (% of data available)	54/73	53/68 (77.9)	1/6	
GAPP 3-6, n (% of data available)	10/73	9/68 (13.2)	2/6	
GAPP ≥7 (% of data available)	9/73	6/68 (8.8)	2/6	
Years of last FU / of death, as appropriate, n (%)				<0.0001
1990s		1 (0.4)	7 (9.7)	0.41 (without 2020s)
2000s		10 (4.3)	17 (23.6)	
2010s		21 (9.1)	34 (47.2)	

	All	Survivors (n=231)	Non-survivors (n=72)	p-value
2010-2014		3	11	
2015-2019		18	23	
2020s		199 (86.1)	14 (19.4)	
of which in 2023 (% of 2020s)		159 (79.9)	1	

Supplementary Table 5. Number of patients with germline tests in the four genetic laboratories over the five decades of follow-up. Initials follow the names of the specialists responsible for the germline analysis and co-authoring this paper. Panel denotes the Neuroendocrine Panel as outlined above.

Lab/Method	1980s	1990s	2000s	2010s	2020s	Total
NGS (V.St., R. L.)		2	5	14	6	27
Panel (H.E.)	1		4	1	1	7
NGS + Panel	1	13	28	45	39	126
Labdia - *RET*, *SDHx*, *VHL* (P.Z.)	4	22	47	28		102
Our lab - *RET* (S.B.)	2	1	4	6		13
NF1 (clinical grounds)		2	1	1		4
No genetic tests	3	13	4	4	0	24
# genetic tests	8	40	90	95	46	279
Total	11	53	94	99	46	303

Supplementary Table 6. Univariate analysis of hazard ratios for overall survival (OAS), disease-specific survival 1 (DSS 1) and disease-specific survival 2 (DSS 2) of all 303 patients. *simpler model with no covariates is correct, n.c.=not calculable, n.d.=not determined (≤5 events only), § calculations without patients with primary metastatic disease.

	Reference level	Overall survival		Disease-specific survival 1		Disease-specific survival 2	
		HR (95% CI)	p-value	HR (95% CI)	p-value	HR (95% CI)	p-value
Age	per year	1.06 (1.04-1.08)	<0.0001	1.02 (0.99-1.06)*	0.22	1.07 (1.05-1.1)	<0.0001
Sex	female	1.9 (1.2-3.0)	0.0008	4.5 (1.5-16.1)	0.01	2.1 (1.1-3.9)	0.01
Location	uPCC						
	PGL	1.5 (0.8-2.7)	0.19	1.4 (0.6-2.8)	0.40	5.2 (1.8-14.5)	0.002
	HNPGL	0.3 (0.05-1.12)	0.14	0.3 (0.01-1.2)	0.17	n.c.	>0.99
Comorb	non-disease						
	CVD	2.4 (1.3-4.6)	0.007	1.3 (0.4-4.0)	0.67	5.1 (2.6-11.0)	<0.0001
	Mal dom	4.4 (2.5-8.3)	<0.0001	n.c.	>0.99	1.2 (0.4-3.1)	0.77
Sympt	non-MACE						
	MACE	4.2 (2.3-7.6)	<0.0001	3.8 (0.8-15.5(	0.07	5.6 (2.6-12.8)	<0.0001
	No sympt	1.7 (0.9-3.0)	0.11	1.02 (0.2-4.2)	0.98	2.0 (0.8-4.6)	0.12
Secr*	yes	0.4 (0.07-1.4)*	0.22	n.c.*	>0.99	3.1 (0.6-75)*	0.27
Tu size	per cm	1.2 (1.09-1.3)	<0.0001	1.5 (1.25-1.7)	<0.0001	1.2 (1.1-1.4)	<0.0001
Prim meta	no	15.5 (7.2-31.5)	<0.0001	41.8 (13.2-129)	<0.0001	20.8 (9.0-46.1)	<0.0001
Meta rec§	no	1.7 (0.8-3.2)	0.12	n.c.*	>0.99	4.8 (2.1-10.7)	0.0001
Nonmeta rec§	no	n.c.*	>0.999	n.c*	>0.99	n.c.	>0.99
All rec §	no	0.7 (0.3-1.3)*	0.24	n.c*	>0.99	1.5 (0.7-3.3)	0.30
PASS	per point	1.2 (1.06-1.33)	0.002	1.3 (0.97-4.9)*	0.7	1.2 (1.05-1.4)	0.005
GAPP	per point	n.d.		n.d.		n.d.	
Genet*	neg	0.7 (0.35-1.1)*	0.15	1.3 (0.3-4.9)*	0.7	0.8 (0.3-1.6)*	0.47
Surg approach	transperit lap						
	open	1.7 (1.009-2.9)*	0.049	17.0 (3.4-310)	0.006	2.8 (1.4-5.9)	0.004
	retroperit lap	0.5 (0.03-2.6)	0.55	n.c.	>0.99	n.c.	>0.99
Surg expert	specialized	1.3 (0.7-2.2)*	0.33	8.4 (2.7-31)	0.0004	1.9 (0.9-3.7)	0.06
Surg dur	per minute	1.003 (1.001-1.005)	0.006	1.004 (0.997-1.009)*	0.13	1.004 (1.00-1.006)	0.02

Supplementary Table 7. Detailed results of germline genetic testing, disease activity (primary metastatic disease, metastatic and nonmetastatic recurrence, as well as no recurrence) and death due to PPGL (disease-specific survival 1), due to CVD, as well as because of malignant and other (mal+) diseases of patients with distinct hereditary disease.

Genetic result	Total	all survivors	all nonsurvivors, n (%)	PPGL death	CVD death	Mal+ death
Negative	184	148	36	5	14	17
prim meta	*5*	*1*	*4*	*2*	*1*	*1*
meta rec	*11*	*6*	*5*	*3*	*1*	*1*
nonmeta rec	*1*	*1*				
no rec	*151*	*129*	*22*		*8*	*14*
rec unknown	*16*	*11*	*5*		*4*	*1*
VHL	14	14	0	0	0	0
prim meta						
meta rec	*1*	*1*				
nonmeta rec	*2*	*2*				
no rec	*11*	*11*				
rec unknown						
NF1	18	12	6	2	0	4
prim meta	*1*		*1*	*1*		
meta rec	*2*	*1*	*1*	*1*		
nonmeta rec	*11*	*10*	*1*			*1*
no rec	*4*	*1*	*3*			*3*
rec unknown						
MEN2A	33	24	9	0	5	4
prim meta						
meta rec	*1*		*1*			*1*
nonmeta rec	*13*	*11*	*2*		*2*	
no rec	*17*	*11*	*6*		*3*	*3*
rec unknown	*2*	*2*				
SDHA	2	2	0	0	0	0
prim meta						
meta rec	*1*	*1*				
nonmeta rec						
no rec	*1*	*1*				
rec unknown						
SDHB	8	6	2	2	0	0
prim meta	*1*		*1*	*1*		
meta rec	*2*	*1*	*1*	*1*		
nonmeta rec	*2*	*2*				
no rec	*3*	*3*				
rec unknown						
SDHC	3	2	1	0	1	0
prim meta	*1*		*1*		*1*	

Genetic result	Total	all survivors	all nonsurvivors, n (%)	PPGL death	CVD death	Mal+ death
meta rec	*1*	*1*				
nonmeta rec						
no rec	*1*	*1*				
rec unknown						
SDHD	15	14	1	0	1	0
prim meta						
meta rec	*1*	*1*				
nonmeta rec	*12*	*11*			*1*	
no rec	*1*	*1*				
rec unknown	*1*	*1*				
FH	1	1	0	0	0	0
prim meta						
meta rec						
nonmeta rec	*1*	*1*				
no rec						
rec unknown						
TMEM127	1	1	0	0	0	0
prim meta						
meta rec						
nonmeta rec						
no rec	*1*	*1*				
rec unknown						
Unknown	24	7	17	6	8	3
prim meta	*4*		*4*	*2*	*2*	
meta rec	*4*		*4*	*4*		
nonmeta rec	*1*		*1*		*1*	
no rec	*12*	*6*	*6*		*3*	*3*
rec unknown	*3*	*1*	*2*		*2*	
Total cohort	303	231	72	15	29	28

Supplementary Table 8. Details of causes of death of 72 nonsurvivors, including age, sex (f=female, m=male), histopathological (Histo) and genetic (Gen) results, decades of first surgery (DOS), decades of death (DOD) and overall survival (OAS) of patients. Age and OAS are given in years at and after first surgery, respectively. Primary metastatic disease was identified in local lymph nodes (Lnn), multiple liver lesions or disseminated. Metastatic recurrences were always disseminated athe the time of death or last FU, respectively. Abbreviations not used in the text: Mal+ = malignant disease and others (not CVD, not COPD), dissem metast = disseminated metastatic, HF = heat failure, AKI = acute kidney insufficiency, AMI = acute myocardial infarction, CAD = coronary artery disease, HF = heart failure, SCD = sudden cardiac death, Ca = carcinoma, AML = acute myeloid leukemia, CKD = chronic kidney disease (-insufficiency).

Pat-ID	Histo	Gen	Age	Sex	DOS	DOD	OAS	Death	Details	Prim metast	Recurrence
288	uPCC	neg	65,51	f	2010s	2020s	5,6825	CVD	Stroke	yes (lungs, bones)	progress, but not cause of death
54	uPCC	neg	66,21	f	2010s	2010s	3,4767	PPGL	dissem metast	no	metast (mult bones, distant Lnn)
135	uPCC	neg	47,46	f	1980s	2000s	16,726	PPGL	dissem metast	no	metast (liver, lungs, mult bones)
252	uPCC	neg	67,21	f	2000s	2020s	19,918	CVD	HF+AKI	no	metast (liver, lungs, distant Lnn)
190	uPCC	neg	49,35	f	2010s	2010s	2	Mal+	Acute panreatitis		
7	uPCC	neg	65,04	m	1990s	2010s	16,471	CVD	AMI		
219	uPCC	neg	44,00	m	2000s	2000s	3,63	CVD	AMI		
235	uPCC	neg	77,16	f	2000s	2020s	12,658	Mal+	Ca/Breast		
120	uPCC	neg	70,68	f	1990s	2010s	19,564	Mal+	Ca/Bronchi		
176	uPCC	neg	52,63	m	1990s	2010s	15,778	Mal+	Ca/Bronchi		
226	uPCC	neg	80,95	m	1990s	2010s	17,447	Mal+	Ca/Colon		
89	uPCC	neg	49,21	f	1980s	2000s	20,46	Mal+	Ca/Pancreas		
26	uPCC	neg	69,80	f	2000s	2000s	1,7892	CVD	CAD		
114	uPCC	neg	76,46	f	2000s	2010s	11,813	CVD	CAD		
100	uPCC	neg	67,08	m	2000s	2010s	13,159	CVD	CAD		
130	uPCC	neg	59,40	m	2000s	2020s	12,394	CVD	CAD		
159	uPCC	neg	71,93	m	2000s	2010s	10,822	CVD	CAD		
216	uPCC	neg	79,93	m	2010s	2010s	4,2633	Mal+	CKD		
146	uPCC	neg	66,23	f	2000s	2010s	12,288	Mal+	Colitis ulcerosa		
41	uPCC	neg	72,74	m	2000s	2010s	7,0167	CVD	HF+AKI		
262	uPCC	neg	56,88	m	2010s	2010s	6,4742	Mal+	M. Parkinson		
214	uPCC	neg	68,64	m	2000s	2010s	13,671	Mal+	Motoneuron disease		
269	uPCC	neg	68,18	f	2000s	2010s	13,666	Mal+	Plasmacytoma		

Pat-ID	Histo	Gen	Age	Sex	DOS	DOD	OAS	Death	Details	Prim metast	Recurrence
40	uPCC	neg	75,11	f	1990s	2000s	4,2217	CVD	Stroke		
104	uPCC	neg	43,17	m	2010s	2020s	8,74	CVD	Stroke		
2	aPGL	neg	44,55	f	2010s	2010s	3,74	Mal+	Ca/Kidney	yes (local Lnn)	progress, but not cause of death
237	aPGL	neg	54,90	f	1990s	2000s	9,7208	PPGL	dissem metast	yes (liver)	progress until death
303	aPGL	neg	76,20	m	2000s	2000s	6,5675	PPGL	dissem metast	no	metast (liver, mult bones)
201	aPGL	neg	49,85	f	2000s	2020s	15,258	Mal+	Ca/Bronchi		
103	aPGL	neg	67,47	f	2000s	2010s	15	CVD	CAD		
270	aPGL	neg	74,33	m	2000s	2000s	2,8442	Mal+	Meningioma		
125	tPGL	neg	55,51	m	2010s	2020s	2,7558	PPGL	dissem metast	yes (liver, distant Lnn)	progress until death
137	cPCC	neg	80,82	f	2010s	2020s	5,9017	Mal+	Ca/Colon		
166	cPCC	neg	46,98	f	2000s	2020s	16,408	Mal+	Ca/Stomach		
170	cPCC	neg	56,48	f	2000s	2010s	9,8467	CVD	CAD		
274	HNPGL	neg	14,61	f	1980s	2020s	33,438	Mal+	AML	no	metast (liver, lungs, mult bones)
189	uPCC	NF1	45,89	m	2010s	2020s	4,6958	PPGL	dissem metast	yes (liver)	progress until death
127	uPCC	NF1	30,28	m	1990s	1990s	0,545	PPGL	dissem metast	no	metast (liver, lungs, mult bones)
115	uPCC	NF1	51,85	m	2000s	2010s	16,959	Mal+	NF1	no	nonmetast (biochem)
56	uPCC	NF1	42,12	f	1990s	2010s	18,625	Mal+	Ca/Pancreas		
273	uPCC	NF1	65,02	m	2000s	2010s	14,913	Mal+	NF1		
151	bPCC	NF1	43,55	m	1990s	2000s	9,5783	Mal+	NF1		
248	uPCC	MEN2A	56,78	f	1990s	2010s	14,381	CVD	AMI	no	nonmetast (PCC contralat)
33	uPCC	MEN2A	33,21	m	1968	2010s	48,268	CVD	CAD	no	nonmetast (PCC contralat)
145	uPCC	MEN2A	33,88	m	2000s	2010s	16,959	Mal+	Asphyxia		
225	uPCC	MEN2A	75,81	f	2000s	2010s	16,038	Mal+	Ca/Pancreas		
136	uPCC	MEN2A	73,11	f	1990s	2010s	20,213	CVD	CAD		
69	uPCC	MEN2A	62,73	m	2010s	2020s	3,5558	CVD	HF+KI		
118	tPGL	MEN2A	38,73	m	1980s	2010s	26,945	Mal+	Ca/Bronchi	no	metast (liver, lungs, mult bones)
230	bPCC	MEN2A	72,05	f	1990s	2000s	7,1642	CVD	AMI		
82	bPCC	MEN2A	56,58	m	2000s	2010s	12,778	Mal+	Ca/Thyroid (MTC)		
51	aPGL	SDHB	14,72	m	2000s	2010s	8,255	PPGL	dissem metast	yes (local Lnn)	progress until death
148	aPGL	SDHB	16,28	f	2000s	2010s	5,4983	PPGL	dissem metast	no	metast (mult bones, distant Lnn)
113	aPGL	SDHC	61,31	f	2000s	2010s	9,8792	CVD	AMI	yes (local Lnn)	progress, but not cause of death
265	HNPGL	SDHD	34,12	m	1980s	2010s	38,028	CVD	SCD	no	nonmetast (HNPGL)
139	uPCC	unknown	66,44	m	1990s	1990s	2,3675	CVD	AMI	yes (local Lnn)	progress, but not cause of death

Pat-ID	Histo	Gen	Age	Sex	DOS	DOD	OAS	Death	Details	Prim metast	Recurrence
142	uPCC	unknown	34,67	m	1990s	1990s	2,4742	PPGL	dissem metast	yes (local Lnn)	progress until death
247	uPCC	unknown	70,72	f	1990s	1990s	6,9317	CVD	Stroke	yes (liver)	progress, but not cause of death
295	uPCC	unknown	33,32	m	2010s	2010s	4,5292	PPGL	dissem metast	no	metast (liver, lungs, brain)
228	uPCC	unknown	54,44	m	1990s	2010s	21,208	PPGL	dissem metast	no	metast (liver, lungs, mult bones)
210	uPCC	unknown	46,47	m	1990s	1990s	2,9367	PPGL	dissem metast	no	metast (mult bones)
77	uPCC	unknown	51,58	m	1980s	1990s	15,244	CVD	CAD	no	nonmetast (PCC ipsilat)
172	uPCC	unknown	84,37	f	2010s	2020s	3,0408	CVD	AMI+Stroke		
92	uPCC	unknown	71,59	f	1990s	1990s	0,6792	Mal+	Ca/Bronchi		
98	uPCC	unknown	48,51	m	1990s	2000s	3,825	Mal+	Ca/Bronchi		
241	uPCC	unknown	44,01	m	2000s	2000s	1,7317	Mal+	Ca/Stomach		
204	uPCC	unknown	73,42	f	2000s	2000s	3,63	CVD	CAD		
90	uPCC	unknown	67,94	m	1990s	2000s	11,913	CVD	CAD		
158	uPCC	unknown	77,76	m	1990s	2000s	7,8408	CVD	CAD		
71	uPCC	unknown	69,05	m	1990s	2000s	8,3917	CVD	Stroke		
292	aPGL	unknown	78,01	m	2010s	2020s	4,7783	PPGL	dissem metast	yes (muscles, bones)	progress until death
29	tPGL	unknown	65,07	m	1990s	2000s	8,5892	PPGL	dissem metast	no	metast (mult bones)

Supplementary Table 9. Characteristics of 12 patients with primary metastatic (1º metast) disease and of 57 patients with recurrent PPGL. Abbreviations are those used in the text. n.a.=not applicable, n.d.= not determined. p-values of survival comparisons relate to patients with recurrent disease only (i.e. not including primary metastatic PPGLs). Cluster 1A included pathogenic germline variants of succinate dehydrogenase subunits A-D (*SDHA-D*) and fumarate hydratase (*FH*), cluster 1B of von Hippel-Lindau (*VHL*) tumor suppressor gene and cluster 2 of rearranged-during-transfection (*RET*) proto-oncogene, neurofibromin 1 (*NF1*) tumor suppressor gene and transmembrane protein 127 (*TMEM127*).

	1º metast	All rec	Surv	Nonsurv	p-value
Age at 1st surgery in years, mean±SD	53.4±17.3	36.5±15.4	32.4±12.4	46.1±17.7	0.002
>36a, n (%)	10 (83.3)	24 (42.1)	13 (32.5)	11 (64.7)	0.04
Female sex, n (%)	5 (41.7)	31 (54.4)	25 (62.5)	6 (35.3)	0.08
Survival					
Recurrence free survival (yrs), mean±SD	n.a.	7.3±7.1	6.9±6.7	8.0±8.3	0.80
Post recurrence survival (yrs), mean±SD	n.a.	10.2±9.6	10.8±9.7	8.7±9.6	0.47
Overall survival (yrs), mean±SD	5.2±2.9	10.5±11.4	17.8±10.5	16.7±13.6	0.61
death from PPGL (disease-specific 1), n (%)	6 (50.0)	9 (52.9)	n.a.	9 (52.9)	
death from CVD, n (%)	4 (33.3)	5 (29.4)	n.a.	5 (29.4)	
death from malignancies+other diseases, n (%)	1 (8.3)	3 (17.6)	n.a.	3 (17.6)	
death from PPGL+CVD (disease-specific 2), n (%)	10 (83.3)	14 (82.4)	n.a.	14 (82.4)	
Disease-specific survival 1 (yrs), mean±SD	5.5±2.9	7.8±6.8	n.a.	7.8±6.8	
CVD survival, mean±SD	6.2±3.1	27.2±15.2	n.a.	27.2±15.2	
Malignancies+other diseases survival, mean±SD	3.74	25.8±8.3	n.a.	25.8±8.3	
Disease-specific survival 2 (yrs), mean±SD	5.8±2.9	14.7±13.9	n.a.	14.7±13.9	
Secretory at first recurrence, n (%)					0.31
yes	n.a.	24 (42.1)	18 (45.0)	6 (35.3)	
no	n.a.	15 (26.3)	12 (30.0)	3 (17.6)	
unknown	n.a.	18 (31.6)	10 (25.0)	8 (47.1)	
Number of recurrences per patient	n.a.				0.50
1 recurrence, n (%)	n.a.	43 (75.4)	28 (70.0)	15 (88.2)	
2 recurrences, n (%)	n.a.	9 (15.8)	8 (20.0)	1 (5.9)	
3 recurrences, n (%)	n.a.	1 (1.8)	1 (2.5)	0	
4 recurrences, n (%)	n.a.	2 (3.5)	2 (5.0)	0	
5 recurrences, n (%)	n.a.	2 (3.5)	1 (2.5)	1 (5.9)	

	1º metast	All rec	Surv	Nonsurv	p-value
Histopathological diagnosis					0.03
PCC group, all, n (%)	6 (50.0)	35 (61.4)	25 (62.5)	10 (58.8)	
uPCC unilateral, n (%)	6	32	22	10	
cPCC unilateral, n (%)	0	0	0	0	
bPCC synchronous, n (%)	0	3	3	0	
PGL group, all, n (%)	6 (50.0)	12 (21.1)	8 (20.0)	4 (23.5)	
aPGL, n (% PGL not HNPGL)	6	10	8	2	
tPGL, n (% PGL not HNNPGL)	0	2	0	2	
mPPGL synchronous	0	0	0	0	
HNPGL	0	10 (17.5)	7 (41.2)	3 (17.6)	
Clinical scenarios at presentation					0.61
Oligosymptomatic group, n (%)	1 (8.3)	15 (26.3)	11 (27.5)	4 (23.5)	
a) Incidental, n (%)	1	8	4	4	
b) Screening, n (%)	0	7	7	0	
Non-MACE symptomatic group, n (%)	7 (58.3)	28 (49.1)	21 (52.5)	7 (41.1)	
c) Adrenergic symptoms, n (%)	5	12	11	1	
d) Uncontrolled hypertension, n (%)	1	5	3	2	
e) Suspicion of secondary hypertension, n (%)	1	3	1	2	
f) Lump in the neck/hearing problems, n (%)	0	8	6	2	
MACE group, all, n (%)	0	8 (14.0)	5 (12.5)	3 (17.6)	
g) Incidental despite prior MACE, n (%)	0	2	1	1	
h) Uncontr hypert despite prior MACE, n (%)	0	1	1	0	
k) MACE, n (%)	0	5	3	2	
Unknown, n (%)	3 (25.0)	6 (10.5)	3 (7.5)	3 (17.6)	
Comorbidities					0.36
No comorbid disease group, n (%)	4 (33.3)	24 (42.1)	17 (42.5)	7 (41.1)	
No comorbid disease, n (%)	2	10	6	4	
≥ 2CV risk factors (CV RF), n (%)	2	14	11	3	
CV disease (CVD), n (%)	6 (50.0)	14 (24.6)	8 (20.0)	6 (35.3)	
Malignancy dominated group, n (%)	1 (8.3)	14 (24.6)	12 (30.0)	2 (11.8)	
Malignant disease without CV RF, n (%)	0	9	7	2	
Malignant disease with CV RF, n (%)	1	4	4	0	

	1º metast	All rec	Surv	Nonsurv	p-value
Other*, n (%)	0	1	1	0	
Malignant disease +CVD, n (%)	1 (8.3)	5 (8.8)	3 (7.5)	2 (11.8)	
Family history					0.02
Positive, n (%)	0	20 (35.1)	18 (45.0)	2 (11.8)	0.09 (pos
of which genetic results positive, n (%)	0	20	18	2	
Cluster 1A, n	0	11	10	1	
Cluster 1B, n	0	0	0	0	
Cluster 2, n	0	9	8	1	
of which genetic results negative, n (%)	0	0	0	0	
Negative, n (%)	7 (58.3)	29 (50.9)	19 (47.5)	10 (58.8)	
of which genetic results positive, n (%)	2	15	12	3	
Cluster 1A, n	1	6	6	0	
Cluster 1B, n	0	2	2	0	
Cluster 2, n	1	7	4	3	
of which genetic results negative, n (%)	3	11	7	4	
of which genetic results unknown, n (%)	2	3	0	3	
Unknown, n (%)	5 (41.7)	8 (14.0)	3 (7.5)	5 (29.4)	
of which genetic results positive, n (%)	1	5	3	2	
Cluster 1A, n	1	3	2	1	
Cluster 1B, n	0	1	1	0	
Cluster 2, n	0	1	0	1	
of which genetic results negative, n (%)	2	1	0	1	
of which genetic results unknown, n (%)	2	1	0	1	
Genetic results					0.0003
Positive, n (%)	3 (25.0)	40 (70.2)	33 (82.5)	7 (41.2)	
Cluster 1A, n (% of positive)	2	20	18	2	0.12 (pos
Cluster 1B, n (% of positive)	0	3	3	0	
Cluster 2, n (% of positive)	1	17	12	5	
Negative, n (%)	5 (41.7)	12 (21.1)	7 (17.5)	5 (29.4)	
Unknown, n (%)	4 (33.3)	5 (8.8)	0	5 (29.4)	
Secretory at first diagnosis, n (%)					0.17
yes	9 (75.0)	33 (57.9)	24 (60.0)	9 (52.9)	
no	0	9 (15.8)	8 (20.0)	1 (5.9)	

	1º metast	All rec	Surv	Nonsurv	p-value
unknown	3 (25.0)	15 (26.3)	8 (20.0)	7 (41.2)	
Surgical approach					0.36
Open, n (%)	10 (83.3)	43 (75.4)	28 (70.0)	15 (88.2)	
Laparoscopic transperitoneal, n (%)	0	10 (17.6)	8 (20.0)	2 (17.8)	
Laparoscopic retroperitoneal, n (%)	0	4 (7.0)	4 (10.0)	0	
Transvaginal	0	0	0	0	
No surgery, including 1 Bx*, n (%)	2 (16.7)	0	0	0	
Duration of surgery, min (mean±SD)	186±129	183±146	183±146	197±61	0.24
Surgical experience					0.16
Specialized, n (%)	5 (41.7)	31 (54.4)	24 (60.0)	7 (41.2)	
Nonspecialized, n (%)	5 (41.7)	20 (35.1)	12 (30.0)	8 (47.1)	
Unknown, n (%)	0	6 (10.5)	4 (10.0)	2 (11.7)	
No surgery, n (%)	2 (16.7)	0	0	0	
Decades of first surgery					0.77
1980s (incl. 1 in 1968*)	0	6 (10.5)	3 (7.5)	3 (17.6)	
1990s	3 (25.0)	15 (26.3)	11 (27.5)	4 (23.5)	
2000s	2 (16.7)	19 (33.3)	14 (35.0)	5 (29.4)	
2010s	4 (33.3)	12 (21.1)	9 (22.5)	3 (17.6)	
2020s	1 (8.3)	5 (8.8)	3 (7.5)	2 (11.8)	
No surgery (1991, 2017)	2 (16.7)	0	0	0	
Tumor size in cm, mean±SD	8.7±3.8	6.2±3.8	4.7±2.8	9.3±3.7	<0.0001
<6cm, n (% of data available)	2/11 (18.2)	26/46 (56.5)	24/33 (72.7)	2/13 (15.4)	0.0007
≥6cm, n (% of data available)	9/11 (81.8)	20/46 (43.5)	9//33 (27.3)	11/13 (84.6)	
PASS score, median (range) of n=17	9 (6-12)		1 (1-16)	4.5 (0-6)	0.36
PASS <4, n (% of data available)	0/4	10/17 (58.8)	8/11 (72.7)	2/6 (33.3)	0.0007
PASS ≥4, n (% of data available)	4/4 (100.0)	7/17 (41.2)	3/11 (27.3)	4/6 (66.7)	
GAPP score, median (range), of	7.5 (6-8)	2 (0-8)	2 (0-8)	n.d.	
GAPP <3, n (% of data available)	0/4	9/14 (64.3)	9/14 (64.3)	n.d.	
GAPP 3-6, n (% of data available)	1/4 (25.0)	4/14 (28.6)	4/14 (28.6)	n.d.	
GAPP ≥7 (% of data available)	3/4 (75.0)	1/14 (7.1)	1/14 (7.1)	n.d.	

	1º metast	All rec	Surv	Nonsurv	p-value
Years of last FU / of death, as appropriate, n (%)					<0.0001
1990s	3 (25.0)	3 (5.3)	0	3 (17.6)	0.99
2000s	1 (8.3)	5 (8.8)	1 (2.5)	4 (23.5)	
2010s	3 (25.0)	11 (19.3)	2 (5.0)	9 (52.9)	
2010-2014	2	5	0	5	
2015-2019	1	6	2	4	
2020s	5 (41.7)	38 (66.7)	37 (92.5)	1 (5.9)	
of which in 2023 (% of 2020s)	1 (20.0)	33 (86.8)	33 (89.2)	0	

Supplementary Table 10. Characteristics of 57 patients with recurrent PPGL, 24 (12 surviving and 12 nonsurviving, abbreviated surv and nonsurv) with metastatic recurrent (abbreviated rec) and of 33 (28 surviving and 5 nonsurviving) with nonmetastatic recurrent PPGL. n.a.=not applicable, n.d.= not determined. Cluster 1A included pathologic germline variants of succinate dehydrogenase subunits A-D (*SDHA-D*) and fumarate hydratase (*FH*), cluster 1B those of von Hippel-Lindau (*VHL*) tumor suppressor gene and cluster 2 of rearranged-during-transfection (*RET*) proto-oncogene, neurofibromin 1 (*NF1*) tumor suppressor gene and transmembrane protein 127 (*TMEM127*).

	Metast rec,	Metast rec,	Nonmetast rec,	Nonmetast rec,	p-value
Age at 1st surgery in years, mean±SD	39.1±14.9	46.4±20.3*	29.6±10.2**	45.5±11.0	0.007<br>0.03 (* vs. **)
>36a, n (%)	6 (50.0)	8 (66.7)	7 (25.0)	3 (60.0)	0.06
Female sex, n (%)	9 (75.0)	5 (41.7)	16 (57.1)	1 (20.0)	0.17
Survival					
Recurrence free survival (yrs), mean±SD	5.6±4.7	6.9±6.3	7.6±7.3	10.5±12.5	0.95
Post recurrence survival (yrs), mean±SD	13.9±10.3	5.6±5.9	9.5±9.3	16.1±13.1	0.18
Overall survival (yrs), mean±SD	19.6±12.6	12.5±10.8	17.0±9.7	26.6±15.6	0.25
death from PPGL (DSS 1), n (%)	n.a.	9 (75.0)	n.a.	0	<0.0001 (DSS 1 vs.
death from CVD, n (%)	n.a.	1 (8.3)	n.a.	4 (80.0)	0.99 (CVS vs. Mal+)
death from malign+other dis, n (%)	n.a.	2 (16.7)	n.a.	1 (20.0)	
death from PPGL+CVD (DSS2), n (%)	n.a.	10 (83.3)	n.a.	4 (80.0)	0.09 (DSS 2)
Disease-specific survival 1 (yrs), mean±SD	n.a.	7.8±6.8	n.a.	n.a.	
CVD survival, mean±SD	n.a.	19.9	n.a.	29.0±16.9	
Malign+other dis survival, mean±SD	n.a.	27.0, 33.4	n.a.	17.0	
Disease-specific survival 2 (yrs), mean±SD	n.a.	9.0±7.5	n.a.	29.0±16.9	0.008
Secretory at first recurrence, n (%)					
yes	5 (41.7)	5 (41.7)	13 (46.4)	1 (20.0)	
no	4 (33.3)	3 (25.0)	8 (28.6)	0	
unknown	3 (25.0)	4 (33.3)	7 (25.0)	4 (80.0)	
Number of recurrences per patient					
1 recurrence, n (%)	11 (91.7)	11 (91.7)	17 (60.7)	4 (80.0)	
2 recurrences, n (%)	1 (8.3)	0	7 (25.0)	1 (20.0)	
3 recurrences, n (%)	0	0	1 (3.6)	0	
4 recurrences, n (%)	0	0	2 (7.1)	0	
5 recurrences, n (%)	0	1 (8.3)	1 (3.6)	0	

	Metast rec,	Metast rec,	Nonmetast rec,	Nonmetast rec,	p-value
Histopathological diagnosis					0.59
PCC group, all, n (%)	7 (58.3)	6 (50.0)	18 (64.3)	4 (80.0)	
uPCC unilateral, n (%)	7	6	15	4	
cPCC unilateral, n (%)	0	0	0	0	
bPCC synchronous, n (%)	0	0	3	0	
PGL not HNPGL group, all, n (%)	4 (33.3)	4 (33.3)	4 (14.3)	0	
aPGL, n (% PGL not HNPGL)	4	2	4	0	
tPGL, n (% PGL not HNNPGL)	0	2	0	0	
mPPGL synchronous	0	0	0	0	
HNPGL	1 (8.3)	2 (16.7)	6 (21.4)	1 (20.0)	
Clinical scenarios at presentation					
Oligosymptomatic group, n (%)	3 (25.0)	4 (33.3)	8 (28.6)	0	
a) Incidental, n (%)	3	4	1	0	
b) Screening, n (%)	0	0	7	0	
Non-MACE symptomatic group, n (%)	4 (33.3)	3 (25.0)	17 (60.7)	4 (80.0)	
c) Adrenergic symptoms, n (%)	2	0	9	1	
d) Uncontrolled hypertension, n (%)	1	1	2	1	
e) Suspicion of secondary hypertension, n (%)	0	1	1	1	
f) Lump in neck/hearing probl, n (%)	1	1	5	1	
MACE group, all, n (%)	3 (25.0)	2 (16.7)	2 (7.1)	1 (20.0)	
g) Incidental desp prior MACE, n (%)	1	1	0	0	
h) Uncontr hypert desp prior MACE, n (%)	0	0	1	0	
k) MACE, n (%)	2	1	1	1	
Unknown, n (%)	2 (16.7)	3 (25.0)	1 (3.6)	0	
Comorbidities					
No comorbid disease group, n (%)	6 (50.0)	5 (41.7)	11 (39.3)	2 (40.0)	
No comorbid disease, n (%)	2	3	4	1	
≥ 2CV risk factors (CV RF), n (%)	4	2	7	1	
CV disease (CVD), n (%)	3 (25.0)	4 (33.3)	5 (17.9)	2 (40.0)	
Malignancy dominated group, n (%)	1 (8.3)	2 (16.7)	11 (39.3)	0	
Malignant disease without CV RF, n (%)	0	2	7	0	
Malignant disease with CV RF, n (%)	1	0	3	0	

	Metast rec,	Metast rec,	Nonmetast rec,	Nonmetast rec,	p-value
Other*, n (%)	0	0	1	0	
Malignant disease +CVD, n (%)	2 (16.7)	1 (8.3)	1 (3.6)	1 (20.0)	
Family history					0.0002
Positive, n (%)	1 (8.3)	0	17 (60.7)	2 (40.0)	
of which genetic results positive, n (%)	1	0	17	2	0.0005 (pos vs neg)
Cluster 1A, n	1	0	9	1	
Cluster 1B, n	0	0	0	0	
Cluster 2, n	0	0	8	1	
of which genetic results negative, n (%)	0	0	0	0	
Negative, n (%)	9 (75.0)	8 (66.7)	10 (35.7)	2 (40.0)	
of which genetic results positive, n (%)	3	1	9	2	
Cluster 1A, n	1	0	5	0	
Cluster 1B, n	1	0	1	0	
Cluster 2, n	1	1	3	2	
of which genetic results negative, n (%)	6	4	1	0	
of which genetic results unknown, n (%)	0	3	0	0	
Unknown, n (%)	2 (16.7)	4 (33.3)	1 (3.6)	1 (20.0)	
of which genetic results positive, n (%)	2	2	1	0	
Cluster 1A, n	2	1	0	0	
Cluster 1B, n	0	0	1	0	
Cluster 2, n	0	1	0	0	
of which genetic results negative, n (%)	0	1	0	0	
of which genetic results unknown, n (%)	0	1	0	0	
Genetic results					<0.0001
Positive, n (%)	6 (50.0)	3 (25.0)	27 (96.4)	4 (80.0)	<0.0001 (pos vs. neg)
Cluster 1A, n (% of positive)	4	1	14	1	
Cluster 1B, n (% of positive)	1	0	2	0	
Cluster 2, n (% of positive)	1	2	11	3	
Negative, n (%)	6 (50.0)	5 (41.7)	1 (3.6)	0	
Unknown, n (%)	0	4 (33.3)	0	1 (20.0)	
Secretory at first diagnosis, n (%)					
yes	7 (58.3)	6 (50.0)	17 (60.7)	3 (60.0)	

	Metast rec,	Metast rec,	Nonmetast rec,	Nonmetast rec,	p-value
no	2 (16.7)	0	6 (21.4)	1 (20.0)	
unknown	3 (25.0)	6 (50.0)	5 (17.9)	1 (20.0)	
Surgical approach					
Open, n (%)	9 (75.0)	11 (91.7)	19 (67.9)	4 (80.0)	
Laparoscopic transperitoneal, n (%)	2 (16.7)	1 (8.3)	6 (21.4)	1 (20.0)	
Laparoscopic retroperitoneal, n (%)	1 (8.3)	0	3 (10.7)	0	
Transvaginal	0	0	0	0	
No surgery, including 1 Bx*, n (%)	0	0	0	0	
Duration of surgery, min (mean±SD)	146±50	193±72	200±173	205±50	
Surgical experience					
Specialized, n (%)	5 (41.7)	5 (41.4)	19 (67.9)	2 (40.0)	
Nonspecialized, n (%)	5 (41.7)	6 (50.0)	7 (25.0)	2 (40.0)	
Unknown, n (%)	2 (16.7)	1 (8.3)	2 (7.1)	1 (20.0)	
No surgery, n (%)	0	0	0	0	
Decades of first surgery					
1980s (incl. 1 in 1968*)	2 (16.7)	0	1 (3.6)	3* (60.0)	
1990s	3 (25.0)	3 (25.0)	8 (28.6)	1 (20.0)	
2000s	4 (33.3)	4 (33.3)	10 (35.7)	1 (20.0)	
2010s	1 (8.3)	3 (25.0)	8 (28.6)	0	
2020s	2 (16.7)	2 (16.7)	1 (3.6)	0	
No surgery (1991, 2017)	0	0	0	0	
Tumor size in cm, mean±SD	6.8±3.7	9.5±3.7	4.3±2.3	8.7±4.5	0.0007
<6cm, n (% of data available)	6 (60.0)	1 (10.0)*	18 (78.3)**	1	0.0009 (* vs. **)
≥6cm, n (% of data available)	4 (40.0)	9 (90.0)	5 (21.7)	2	0.001
PASS score, median (range) of n=17	4 (0-16)	3.5 (0-6)	1 (1-7)	5 (4-6)	0.07
PASS <4, n (% of data available)	1/3 (33.3)	2/4 (50.0)	7/8	0/2	
PASS ≥4, n (% of data available)	2/3 (66.7))	2/4 (50.0)	1/8	2/2	
GAPP score, median (range), of	4 (1-8)	n.d.	2 (0-5)	n.d.	
GAPP <3, n (% of data available)	1/4 (25.0)	n.d.	8/10 (80.0)	n.d.	
GAPP 3-6, n (% of data available)	2/4 (50.0)	n.d.	2/10 (20.0)	n.d.	
GAPP ≥7 (% of data available)	1/4 (25.0)	n.d.	0	n.d.	

	Metast rec,	Metast rec,	Nonmetast rec,	Nonmetast rec,	p-value
Years of last FU / of death, as appropriate, n (%)					<0.0001
1990s	0	2 (16.7)	0	1 (20.0)	0.99 (without 2020s)
2000s	0	3 (25.0)	1 (3.6)	1 (20.0)	
2010s	0	6 (50.0)	2 (7.1)	3 (60.0)	
2010-2014	0	3	0	2	
2015-2019	0	3	2	1	
2020s	12 (100.0)	1 (8.3)	25 (89.3)	0	
of which in 2023 (% of 2020s)	11 (91.7)	0	22 (88.0)	0	

Supplementary Table 11. Number of deaths due to PPGL, CVD, as well as malignant and other diseases (Mal+) during the study period. Nonsurvivors died with comparable relative frequency from PPGL, CVD, or malignant disease in the 1990s, the 200s, the 2010s and the 2020s.

	PPGL	CVD	Mal+	Total	p-value
1990s	3	3	1	7	0.40
2000s	4	8	5	17	
2010s	5	17	18	34	
2020s	3	6	5	14	
Total	15	29	28	72	

Supplementary Table 12. Predictive factors for overall survival (OAS), disease-specific survival 1 (DSS 1) and disease-specific survival 2 (DSS 2) by Cox regression analysis for 291 patients with primary nonmetastatic PPGL (e.g. excluding 12 patients with metastatic disease at first diagnosis). The predictive factors for recurrence free survival (RFS) were the same as for the entire cohort (see Table 3). *HR for OAS of tumor size and of metastatic recurrence are from two different models keeping the other parameters constant. These two models included equal numbers of parameter estimates and number (#) of parameters per event. The significance of the other parameters became not different, when metastatic recurrence was replaced by tumor size (OAS). Given the small number of events (n=9 deaths from PPGL), the predictive model for DSS1 precluded the analysis of more than one factor and all events (deaths from PPGL) could be predicted by metastatic recurrence (perfect HR).

	Reference level	OAS		DSS 1		DSS 2	
		HR (95% CI)	p-value	HR (95% CI)	p-value	HR (95% CI)	p-value
Age	per year	1.07 (1.04-1.1)	<0.0001				
Sex	female	2.7 (1.5-5.0)	0.001				
Comorbidity	No disease						
	CVD group vs Ref	1.8 (0.7-4.3)	0.20				
	Mal dis group vs Ref	4.6 (2.0-11.0)	0.0004				
	Mal+CVD group vs Ref	1.03 (0.3-3.0)	0.96				
Symptoms	Non-MACE Sympt						
before diagnosis	*MACE*	3.4 (1.4-8.8)	0.01		0.043	5.7 (1.8-20.0)	0.004
	Oligosymptomatic	1.8 (0.9-3.7)	0.09		0.16	4.1 (1.4-13.2)	0.01
Secretory	yes						
Tumor size	per cm	1.1 (0.97-1.2)	0.13*				
Metast recurrence	no	1.6 (0.6-3.5)	0.29*	perfect	n.d.	2.9 (1.02-7.8)	0.04
Genetics	neg						
Genetic cluster	neg						
	Cluster 1A						
	Cluster 1B						
	Cluster 2						
		# param/event = 8*		# param/event = 5, and 3**		# param/event = 8	

Supplementary Table 13. Predictive factors for recurrence-free survival of patients with metastatic (n=24) and nonmetastatic (n=33) recurrences of PPGL by Cox regression. *simpler model with no covariates is correct, n.c.=not calculable, n.d.=not determined (≤5 events only), § second models including age instead of surgical expertise as the second dependent variable.

	Univariate					Multivariate				
	Reference	Metast rec		Nonmetast rec		Reference	Metast rec		Nonmetast rec	
		HR (95% CI)	p-value	HR (95% CI)	p-value		HR (95% CI)	p-value	HR (95% CI)	p-value
Age	per year	0.97 (0.95-1.001)*	0.054	0.92 (0.90-0.95)	<0.0001	per year			0.97 (0.93-1.001)§	0.06
Sex	female	1.2 (0.5-2.6)*	0.70	1.1 (0.5-2.4)*	0.77					
Location	PCC									
	PGL	1.04 ((0.3-2.8)	0.95	0.98 (0.3-2.3)	0.96					
	HNPGL	1.1 (0.06-5.4)	0.92	1.2 (0.2-4.2)	0.83					
Comorb*	No disease									
	CVD	1.8 (0.7-4.6)*	0.23	2.0 (0.8-4.6)*	0.11					
	Mal dom	1.2 (0.4-3.1)*	0.73	1.4 (0.4-3.7)*	0.57					
Sympt	Non-									
	MACE	4.7 (1.4.-15.4)	0.01	0.7 (0.1-2.3)*	0.58					
	No	1.6 (0.6-4.8)	0.36	0.9 (0.4-2.0)*	0.76					
Secr	yes	2.5 (0.4-9.2)*	0.22	1.5 (0.3-5.3)*	0.56					
Tu size	per cm	1.4 (1.2-1.6)	<0.0001	0.8 (0.6-0.97)	0.04	per cm	1.3 (1.13-1.5)	0.0002	0.8 (0.6-0.97)	0.04
PASS	per point	1.2 (1.01-1.45)	0.01	0.95 (0.6-1.2)*	0.78					
GAPP	per point	n.d.		0.99 (0.7-1.4)*	0.99					
Genet	neg	1.9 (0.7-4.6)*	0.17	50.2 (10.7-895)	<0.0001					
Cluster	neg									
	Cluster	-		149 (30-2703)	<0.0001	*neg*				
	Cluster	-		18.5 ((1.8-406)	0.51	*Cluster1*			71 (14.1-1288)	<0.0001
	Cluster	-		31.4 (6.0-576)	0.001	*Cluster 2*			29.5 (5.4	0.001
Surg approach	transperit									
	open	11.4 (3.9-48.6)	<0.0001	4.1 (1.7-11.3)	0.003					
	retro-	2.9 (0.1-23.9)	0.36	5.5 (1.1-22.6)	0.02					
Surg expert	specialized	5.0 (2.1-12.1)	0.0002	1.4 (0.5-3.2)*	0.47	specialized	3.8 (1.13-1.5)	0.005		
Surg dur	per minute	1.003 (0.999-1.01)*	0.40	1.004 (0.999-	0.08					

Supplementary Table 14. Comparison of multivariable analyses of predictive factors for overall survival (OAS), disease-specific survival 1 (DSS 1) and DSS 2 by Cox regression (presenting hazard ratios, HR) and logistic regression (presenting odds ratios, OR) including 95% confidence intervals (95% CI). The conventionally accepted rule of thumb that there should be a minimum of 10 events per parameter studied is given for every HR and OR. However, this remains a controversial topic because simulations with 5-9 events per parameter were comparable to 10-16 (43). Exchanging location (or other dependent variables) for metastatic disease at first diagnosis led to statistical overfitting for DSS1 and was not better than the simpler model containing no covariates (the crossed-out HR and p-values for location ** and diagnosis *** are for illustrative purposes only).

		OAS				DSS 1					DSS 2			
	**Reference**	**HR (95% CI)**	**p-value**	**OR (95% CI)**	**p-value**	**HR (95% CI)**	**p-value**	**Reference**	**OR (95% CI)**	**p-value**	**HR (95% CI)**	**p-value**	**OR (95% CI)**	**p-value**
Age (yrs)	per year	1.05 (1.03-1.07)	<0.0001	1.06 (1.03-1.09)	<0.0001			per year	~~0.11 (0.003-0.20)~~**	0.91	1.06 (1.03-1.09)	<0.0001	1.05 (1.02-1.08)	<0.0001
Sex	female										2.6 (1.2-5.6)	0.01		
Location	PCC													
	PGL vs Ref					~~2.5 (0.5-9.0)~~ **	0.20							
	HNPGL vs Ref					~~0.2 (0.02-1.3)~~ **	0.13							
Comorbidity	No disease group													
	CVD group vs. Ref													
	Mal dis group vs Ref													
	Mal+CVD group vs Ref													
Diagnosis	Non-MACE Symptoms							No and non-MACE Sympt						
	MACE group vs Ref	5.0 (2.0-13.1))	0.0007	6.5 (2.5-17.6)	0.0002	~~3.6 (0.73-14.6)~~ ***	0.08	*MACE vs Ref*	~~1.2 (0.4-12.4)~~***	0.22	4.3 (1.6-12.4)	0.003	3.1 (1.7-9.6)	0.002
	Oligosympt group vs Ref	2.8 (1.3-6.7)	0.01	3.2 (1.5-7.4)	0.005	~~1.02 (0.2-4.1)~~ ***	0.98							
Tumor size (cm)	per cm	1.09 (0.97-1.2)	0.13	1.2 (1.1-1.4)	0.001				1.6 (1.3-2.1)	0.0001			1.3 (1.1-1.6)	0.0007
Meta at 1st diagnosis	no meta	17.3 (5.9-49.1)	<0.0001	70 (19-2970)	<0.0001	41.8 (13.2-129)*	<0.0001		162 (23.8-3280)	<0.0001	23.3 (8.1-62.5)	<0.0001	113 (17-2338)	<0.0001
RFS (yrs)	per year													
Genetics	negative													
	Cluster I vs Ref	0.6 (0.13-2.0)	0.46	1.3 (0.2-4.8)	0.76									
	Cluster II vs Ref	1.3 (0.6-2.5)	0.52	4.3 (1.8-10.7)	0.001									
	OR>1: lower survival	#deaths/#parameters=10.4		#deaths/#parameters=10.4		#deaths/#parameters=15			#deaths/#parameters=7.5		#deaths/#parameters=10.8		#deaths/#parameters=10.8	
	OR<1: better survival					(5 incl location** or MACE***)			(3 incl. Age** or MACE***)					
